# Stakeholders’ views on drug development: the congenital disorders of glycosylation community perspective

**DOI:** 10.1186/s13023-022-02460-0

**Published:** 2022-07-30

**Authors:** Maria Monticelli, Rita Francisco, Sandra Brasil, Dorinda Marques-da-Silva, Tatiana Rijoff, Carlota Pascoal, Jaak Jaeken, Paula A. Videira, Vanessa dos Reis Ferreira

**Affiliations:** 1 Department of Biology, Università degli Studi di Napoli “Federico II”, 80126 Naples, Italy; 2 CDG & Allies — Professionals and Patient Associations International Network (CDG & Allies-PPAIN), Department of Life Sciences, School of Science and Technology, NOVA University Lisbon, 2819-516 Caparica, Portugal; 3 UCIBIO – Applied Molecular Biosciences Unit, Department of Life Sciences, School of Science and Technology, NOVA University Lisbon, 2819-516 Caparica, Portugal; 4 Associate Laboratory i4HB , Institute for Health and Bioeconomy, School of Science and Technology, NOVA University Lisbon, 2819-516 Caparica, Portugal; 5 Portuguese Association for Congenital Disorders of Glycosylation (CDG), Department of Life Sciences, School of Science and Technology, NOVA University Lisbon, 2819-516 Caparica, Portugal; 6 LSRE-LCM – Laboratory of Separation and Reaction Engineering – Laboratory of Catalysis and Materials, Escola Superior de Tecnologia e Gestão, Instituto Politécnico de Leiria, 2411-901 Leiria, Portugal; 7 ALiCE – Associate Laboratory in Chemical Engineering, Faculty of Engineering, University of Porto, Rua Dr. Roberto Frias, 4200-465 Porto, Portugal; 8 CDG Swiss Association, Meyrin, Switzerland; 9 Department of Development and Regeneration, Centre for Metabolic Diseases, KU Leuven, Leuven, Belgium

**Keywords:** Drug development, People-centricity, Congenital disorder(s) of glycosylation (CDG), Electronic (e-)survey, Patient-reported outcome measures

## Abstract

**Background:**

Congenital disorders of glycosylation (CDG) are a large family of rare genetic diseases for which therapies are virtually nonexistent. However, CDG therapeutic research has been expanding, thanks to the continuous efforts of the CDG medical/scientific and patient communities. Hence, CDG drug development is a popular research topic. The main aim of this study was to understand current and steer future CDG drug development and approval by collecting and analysing the views and experiences of the CDG community, encompassing professionals and families. An electronic (e-)survey was developed and distributed to achieve this goal.

**Results:**

A total of 128 respondents (46 CDG professionals and 82 family members), mainly from Europe and the USA, participated in this study. Most professionals (95.0%) were relatively familiar with drug development and approval processes, while CDG families revealed low familiarity levels, with 8.5% admitting to never having heard about drug development. However, both stakeholder groups agreed that patients and families make significant contributions to drug development and approval. Regarding their perceptions of and experiences with specific drug development and approval tools, namely biobanks, disease models, patient registries, natural history studies (NHS) and clinical trials (CT), the CDG community stakeholders described low use and participation, as well as variable familiarity. Additionally, CDG professionals and families shared conflicting views about CT patient engagement and related information sharing. Families reported lower levels of involvement in CT design (25.0% declared ever being involved) and information (60.0% stated having been informed) compared to professionals (60.0% and 85.7%, respectively). These contrasting perceptions were further extended to their insights and experiences with patient-centric research. Finally, the CDG community (67.4% of professionals and 54.0% of families) reported a positive vision of artificial intelligence (AI) as a drug development tool. Nevertheless, despite the high AI awareness among CDG families (76.8%), professionals described limited AI use in their research (23.9%).

**Conclusions:**

This community-centric study sheds new light on CDG drug development and approval. It identifies educational, communication and research gaps and opportunities for CDG professionals and families that could improve and accelerate CDG therapy development.

**Supplementary Information:**

The online version contains supplementary material available at 10.1186/s13023-022-02460-0.

## Background

Congenital disorders of glycosylation (CDG) are a rapidly growing family of rare genetic diseases caused by biosynthetic defects in the glycosylation machinery [1, 2]. Currently, more than 160 CDGs have been identified [3]. CDGs include defects in N-glycosylation, O-glycosylation, glycosylphosphatidylinositol (GPI)-anchor synthesis, glycolipid glycosylation and other glycosylation disorders. Due to their underlying genetic and biochemical variability and the essential physiological roles of glycans and glycoconjugates, CDG have a broad spectrum of clinical phenotypes [3, 4]. They typically present as multiorgan diseases, ranging from mild to severe, encompassing neurological, gastrointestinal, immune and skeletal involvement [5–9].

Most CDGs still lack targeted therapies, and treatment is mainly limited to symptom management. However, a few CDGs can benefit from more specific approaches, namely dietary supplementation and organ transplantation. Indeed, tremendous progress has been made in the management of CDGs, and more recently, therapeutic research has been expanding with chaperones, gene therapy, and drug repurposing, among others [10–16]. A list of the treatments currently under development or already approved is available on the World CDG platform [17].

Nevertheless, despite these remarkable advances and the enormous efforts of the scientific community and the patient advocacy movement to fuel therapeutic research for CDG, there is still an enormous gap to be bridged. Clinical trial (CT) operational and scientific inefficiencies are hurdles that we must identify and address to advance drug development. No doubt, stakeholders must collaborate to push this goal to expedite clinical research and efficiently improve patient care. Consequently, drug discovery and development in CDG are currently popular research topics [10, 11].

In this work, and as part of strategies to minimise delays in patient access to innovative medicines, we explored the views, knowledge and experiences of the CDG community in drug development and approval. For this purpose, we used the privileged networking, research and education platform, the World Conference on CDG for Families and Professionals [12]. To effectively achieve this goal and ensure representative participation among the CDG community, an electronic (e-)survey, adapted to the target audience, was developed and administered. The specific aims of this study were the following.

- Capture a comprehensive and inclusive vision of the CDG drug development and approval process involving different stakeholder groups. In addition to focusing on the diverse phases (and associated tools) of these processes, significant emphasis was placed on clarifying of the role and involvement of patients and family members in therapeutic research.
- Identify knowledge, participation and communication gaps, and map therapeutic research preferences and priorities among the CDG community stakeholders.
- Ultimately, steer ongoing and future research to optimise efforts, obtain faster results and develop approaches that cater to the differential needs and expectations of the CDG community stakeholders.

## Methods

### Developing, piloting and refining the “Assessing CDG needs and solutions for future therapies” e-survey for CDG families and professionals

The “Assessing CDG needs and solutions for future therapies” e-survey was an author-built online questionnaire designed to assess the views, experiences and knowledge of the CDG community regarding drug development and approval. Additionally, the e-survey was created to guide the development of the agenda of the 4th World Conference on CDG for Families and Professionals, converting the conference into a fully community-centric event. The e-survey included a total of 68 questions and was available in English. Questions were adapted to the two stakeholder groups, CDG professionals and CDG families. A pilot phase, which included 8 CDG community members (6 professionals and 2 family members), was conducted to test and improve the understandability and content.

The e-survey was built in and administered through the SurveyMonkey platform (http://www.surveymonkey.net—Copyright#1999–2021 SurveyMonkey). Internet protocol (IP) identifier recording was blocked to ensure respondents’ anonymity. Multiple-entry restriction features were activated to avoid participant duplication. Various question formats, namely multiple choice, matrix, and open-ended questions were used. Before final submission, respondents could review and edit their answers using the ‘Prev.’ button. SurveyMonkey’s logic feature was added to specific questions to reduce the participation burden [13, 14]. A copy of each survey is provided as Additional file 12 (version adapted to professionals) and Additional file 13 (version adapted to families).

Ethical approval for this study was granted by the ethics committee of the Faculty of Psychology, University of Lisbon (reference 1.14/8/2018-19). Electronic informed consent was obtained from all participants.

### Survey recruitment and distribution

The e-survey was launched online before the 4th World Conference on CDG—on May 16, 2019—and remained open to participation for 62 days. The survey was distributed throughout social media and via email messaging to maximise participation, ensure targeted recruitment and overcome geographical barriers. The social media channels of the Portuguese Association for CDG (APCDG), a nonprofit with a long-standing presence in the community and the main organiser of the World Conference on CDG for Families and Professionals, were also used. The email list of the registered participants to the 4th edition for the conference was utilised to distribute the survey.

To guarantee the quality of the recruitment materials, the health communication agency *Miligrama - Comunicação em Saúde* integrated the project and assisted in creating social community posts and messages. An example of the survey recruitment posts is shown in Additional file 1: Figure S1. Because the measures undertaken to certify recruitment and distribution were specifically targeted to different stakeholders of the CDG community, ‘random’ participation of other participants (non-CDG community stakeholders) was considered very unlikely.

## Results

### Participant characteristics

**Fig. 1:**
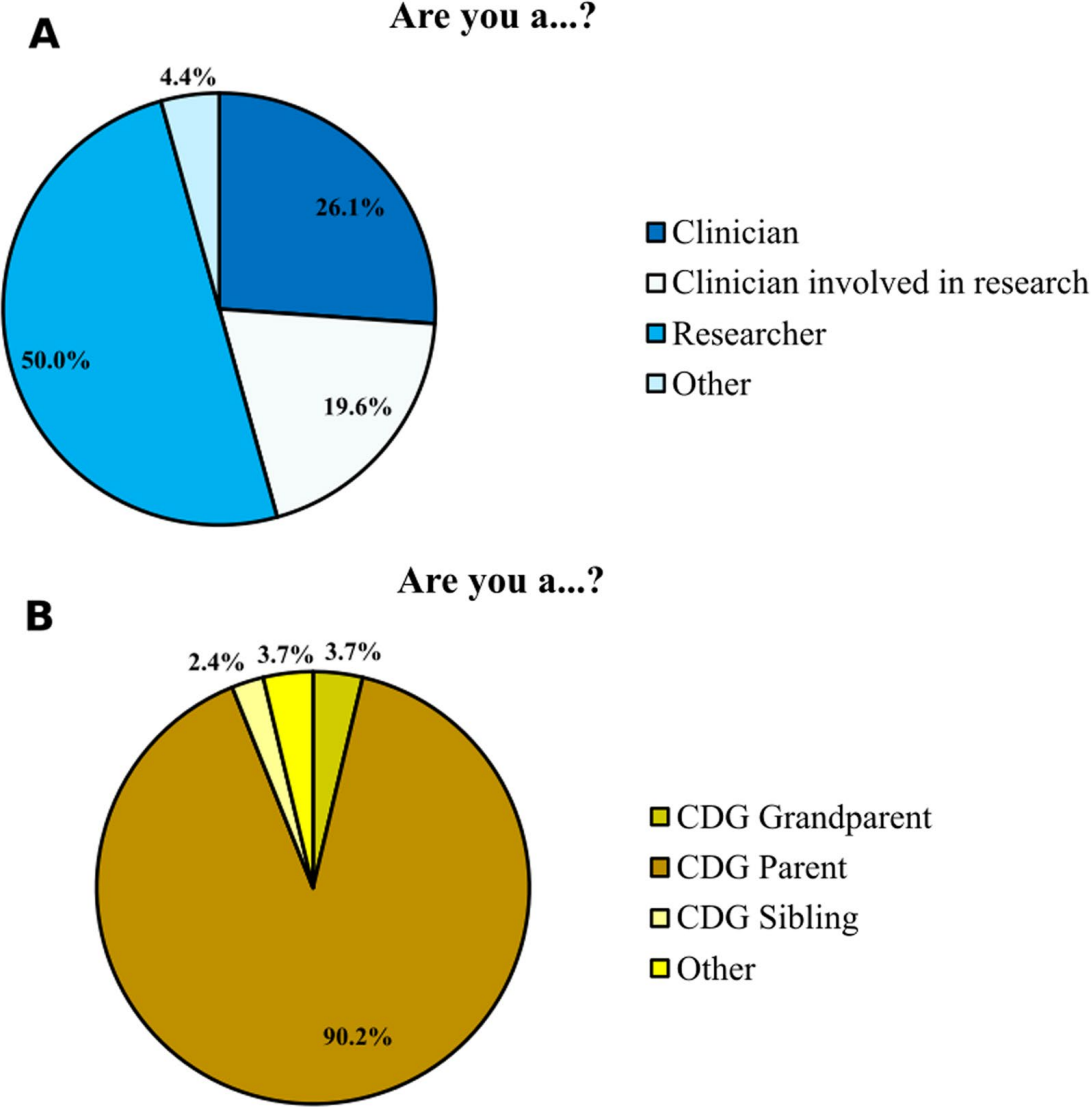
E-survey participants distribution. CDG professionals (**A**) and families (**B**) by category, respectively

A total of 128 individuals (46 CDG professionals and 82 relatives of patients) completed and returned the questionnaire designed for this study. Fifty percent of those surveyed were researchers, and the majority (90.2%) of the family members were parents of CDG patients (Fig. 1). Participation in the survey was spread across a broad geographical area, with the most significant number of participants coming from Europe and the United States. The professionals were mainly from Portugal, the USA and Spain, while family members were mainly from the USA, Australia and Spain (Additional file 9: Table S1).

### CDG drug development and approval awareness

Drug development and approval are complex, stepwise processes involving multiple stakeholders with different roles, powers, interests, and influences. To assess the CDG professionals’ and families’ general awareness level of this topic, we inquired about their familiarity with the process. Overall, more than 95.0% of professionals were familiar with drug development procedures, although only 10.9% reported being very familiar with the entire process (Table 1). In particular, only 23.9% of the professional stakeholders were involved in clinical drug development for CDG (Additional file 2: Figure S2, Panel A). Consequently, we proceeded to explore further the difficulties experienced by professionals regarding therapy research for CDG. The two significant difficulties identified were funding (76.1%) and lack of interest from pharmaceutical companies (65.2%) (Additional file 3: Figure S3). The e-survey revealed that professionals identified public funding as a major source (Additional file 4: Figure S4).

**Table 1:** Drug development and approval process awareness among CDG professionals and families

	Professionals	Families	
	Are you familiar with the clinical drug development and approval process? (*n* = 46)	How familiar are you with the clinical drug development process? (*n* = 82)	How familiar do you think you are with the drug approval process? (*n* = 82)
Very familiar	10.9%	4.9%	6.1%
Familiar	39.1%	9.8%	9.8%
Slightly familiar	45.7%	39.02%	42.68%
Not familiar	4.35%	35.4%	39.0%
I don’t know	0%	2.4%	0%
I have never heard about	NA	8.5%	2.4%

In contrast, when we asked families about their awareness of drug development, only 53.8% stated they were somewhat familiar with this process, while 8.5% had never heard about it (Table 1) (Additional file 9: Table S1).

About authorisation by the regulatory agencies, approximately two-thirds of the professionals (67.4%) stated that patients have a role to play in it, particularly in increasing disease awareness (87.1%) and lobbying for drug therapy approval (80.6%) (Additional file 10: Table S2). In addition, CDG families showed overall poor familiarity with the drug approval process, and most family stakeholders (79.3%) believed that patients can contribute to drug approval (Table 2).

**Table 2:** Views and perceptions of families’ involvement in the drug approval process among CDG professionals and families

	Professionals (*n* = 46)	Families (*n* = 82)
	Do you think patients have a role to play in the drug approval process?	
Yes	67.4%	79.3%
No	15.2%	11.0%
I don’t know	17.4%	9.8%

Despite their different awareness levels, CDG professional and family stakeholders upheld that patients and families have a relevant role in drug development and approval.

To further understand and detail the CDG community stakeholders’ awareness, participation and involvement throughout the drug development and approval process, we investigated their views and experiences regarding biobanks, disease models, patient registries, NHS and CT.

#### Biobanks and disease model awareness

Biobanks (i.e., sample and associated data repositories) and disease models (in vitro, in vivo and computational models) are vital key tools for drug development. In particular, they are of great relevance in preclinical research. Because patients and their families are less involved in these research stages, specific related questions were tailored to the two stakeholder groups. The e-survey confirmed that CDG families were either unaware (11.0%) or uncertain (62.2%) of what biobanks were (Additional file 5: Figure S5, Panel A). Only a minority of professionals (34.8%) reported using biobanks in their CDG therapeutic research (Additional file 2: Figure S2, Panel B). When confronted with the reasons for this low use, many professionals mentioned being unaware of any CDG biobanks (45.9%). Additionally, 13.5% claimed that biobanks did not apply to their research, and 2.7% said that they never felt the need to use them. The remaining 45.9% did not specify the underlying reasons for their limited biobank usage (*not shown*).

When professionals were asked about the barriers to implementing CDG biobanks, most (65.2%) revealed that the main obstacle was the high cost of their implementation and maintenance. Additionally, more than 50.0% identified bureaucracy and lack of interest from professionals in sharing samples (58.7%), as well as sample dispersion (i.e., the existence of small sample collections in each laboratory) (54.3%), as essential barriers to biobank development (Additional file 6: Figure S6).

Similarly, professionals reported low CDG disease model use (45.6%) (Additional file 2: Figure S2, Panel C). Professionals were also asked to share the types of CDG models that they resorted to in their CDG therapeutic research projects. Primary and immortalised patient-derived cell lines were identified as the most common models (90.5% and 57.1%, respectively), followed by commercial cell lines (42.9%). Less commonly used models included induced pluripotent stem cells, as well as yeast (*S. cerevisiae*) and mice (14.3%, each), followed by fish (*D. rerio*, 9.5%), worm (*C. elegans)* and fly (*D. melanogaster)* models (4.8%, each) (Additional file 7: Figure S7).

CDG families showed mixed disease model awareness levels. On the one hand, more than half (53.7%) were familiar with the difference between in vitro and in vivo disease models. On the other hand, only 26.8% of the family participants were familiar with the available disease models for CDG therapeutic discovery (Additional file 5: Figure S5, Panels C and D).

**Fig. 2:**
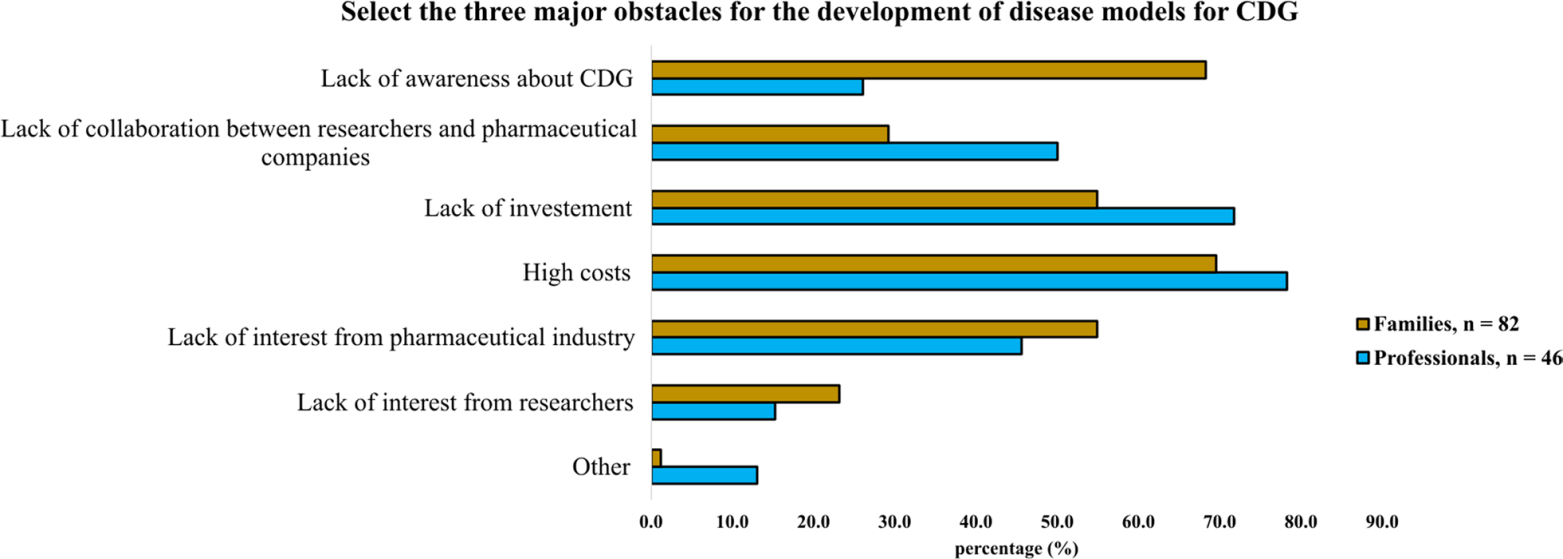
Obstacles to CDG disease model development according to CDG professionals and families

Due to their low use among professionals and low awareness among families, participants were asked to share their opinions about the obstacles to CDG disease model development (Fig. 2). Although funding-related challenges (high costs and lack of investment) were pinpointed by both stakeholder groups (78.3% and 71.7% of the professionals; 69.5% and 54.9% of the families, respectively) as significant barriers, some divergent views emerged. While professionals more frequently saw lack of collaboration between researchers and pharmaceutical companies as an obstacle (50.0% of the professionals in contrast to only 29.3% of the families), lack of CDG awareness was substantially more recognised by CDG families (68.3%) than by professionals (26.1%) as detrimental to CDG disease model creation.

Globally, low biobank and disease model use by professionals and awareness, mostly among CDG families, were described. Both stakeholder groups reported financial reasons as a key obstacle to their development and implementation.

#### CDG community experience with patient registries and natural history studies

Patient registries are critical clinical and health-related data repositories across several aspects of therapeutic research, from biomarker discovery to patient identification and clinical study recruitment. Natural history studies (NHS) collect patient-related information over a finite period of time to investigate the development of the disease in the absence of any therapeutic intervention. Given their importance for drug development and approval, we assessed CDG professionals’ and families’ awareness and experience with patient registries and NHS.

**Fig. 3:**
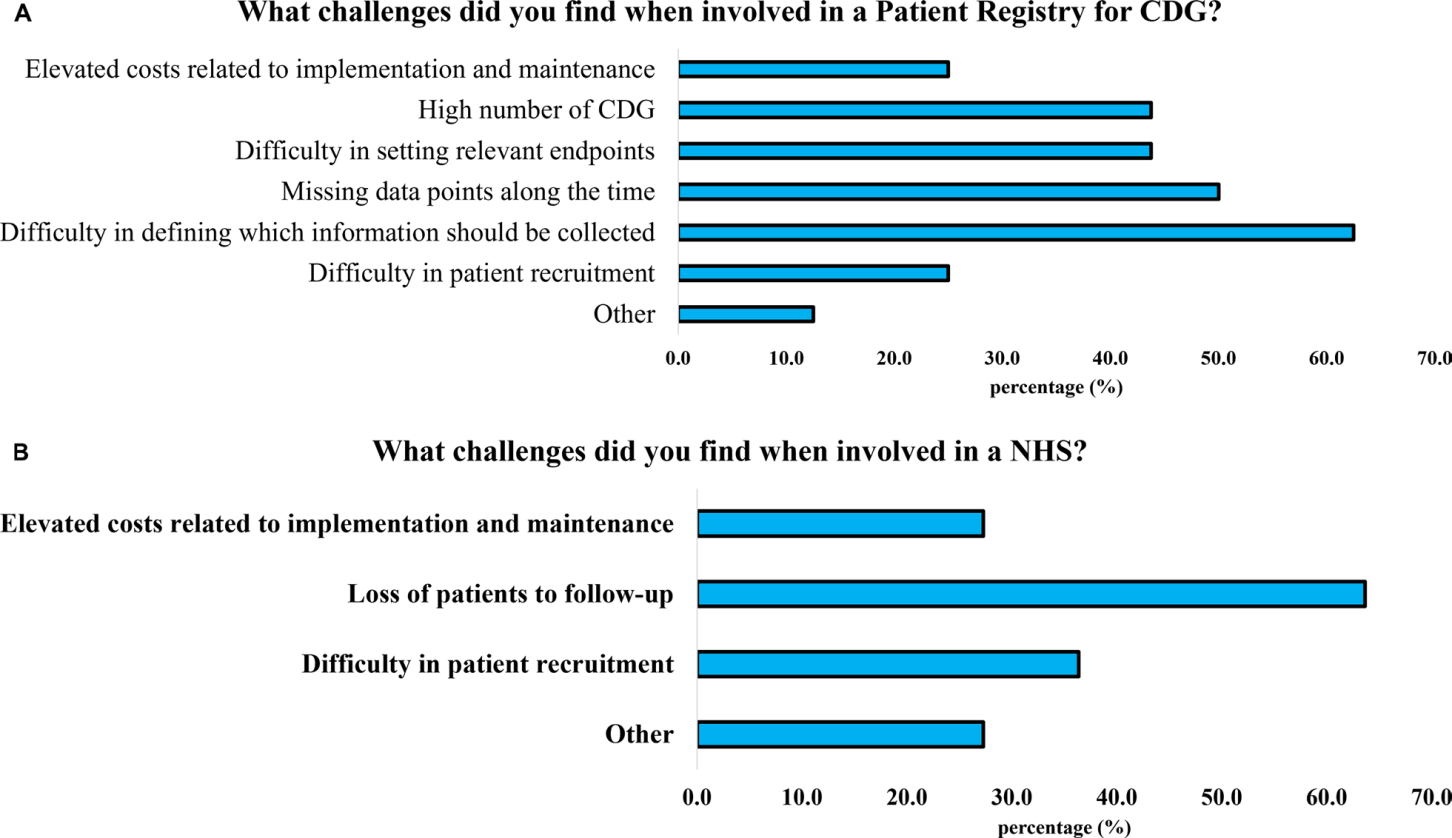
Major challenges faced by CDG professionals in patient registries (n = 16, **A**) and NHS (n = 11, **B**)

Regarding professionals, only a few reported ever being involved in a CDG patient registry (34.8%) or NHS (23.9%) (Table 3). Interestingly, when asked about challenges related to CDG patient registries and NHS, professionals emphasised the difficulties of data collection and management, particularly underscoring the “difficulty in defining which information should be collected” (62.5% for registries) and “loss of patients to follow-up” (63.6% for NHS). Additionally, regarding patient registries, the difficulty in defining which information to collect was identified as a significant issue by the CDG professional community (62.5%) (Fig. 3).

**Table 3:** Involvement of CDG professionals and families in patient registries and NHS

Professionals		Families	
	Are you or have you been involved in a patient registry for CDG? (*n* = 46)		Have your clinical data ever been collected for a patient registry? (*n* = 82)
Yes	34.8%	Yes	40.2%
No	54.3%	No	29.3%
No, and to the best of my knowledge, there are none for CDG	10.9%	I don’t know	30.5%

Professionals		Families	
	Have you ever been involved in a NHS for CDG? (*n* = 46)		Have you ever participated in a NHS? (*n* = 50)
Yes	23.9%	Yes	22.0%
No	67.4%	No	76.0%
No, and to the best of my knowledge, there are none for CDG	8.7%	I don’t know	2.0%

We began by evaluating their patient registry and NHS knowledge and awareness levels for families. Family participants showed greater familiarity with patient registries (64.6%) (Additional file 8: Figure S8, Panel C) than with NHS (50.0%) (Additional file 8: Figure S8, Panel B). In line with these results, 40.2% of families declared knowing that the patient’s data had been collected for a patient registry, while only 22.0% had ever participated in an NHS (Table 3).

**Fig. 4:**
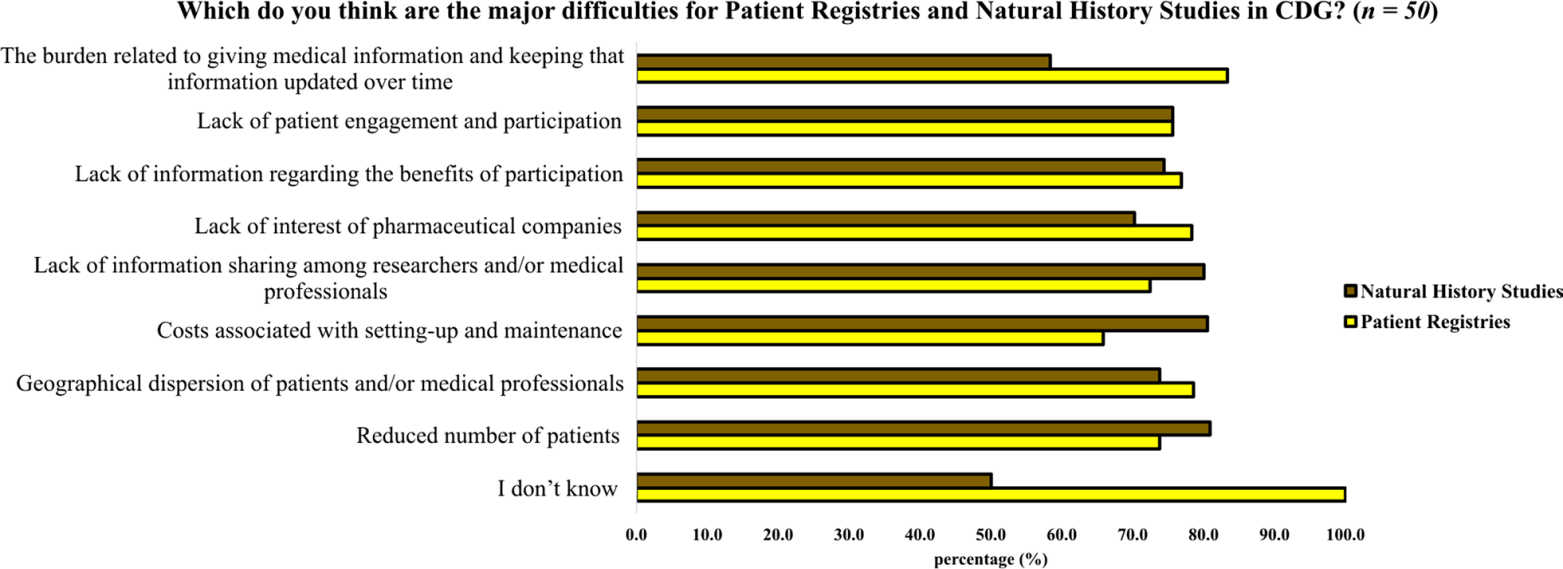
Obstacles identified by CDG families in the development of patient registries and NHS

Although most family participants perceived of patient registries and NHS as being two different drug development tools, they pointed out many similarities when asked to identify the major difficulties related to patient registries and NHS (Fig. 4). Only two significant distinct aspects between registries and NHS stood out. Firstly, the costs associated with setup and maintenance were more commonly associated with NHS (80.5%). Secondly, the burden related to providing medical information and keeping that information updated over time that, in turn, was more prevalent concerning registries (83.3%).

Although having relatively limited experience with patient registries and NHS, CDG professionals and families also revealed higher familiarity and experience with the former. Additionally, the most common patient registries and NHS challenges identified by the CDG community were related to data management and costs.

#### CDG community views and perceptions about patient engagement in and information sharing for CTs

CTs are the last phase of drug development before therapy approval and market access. Only approximately one-third (30.4%) of CDG professionals reported ever being involved in the development of a CT for CDG; of these respondents, more than 60.0% had engaged patient representatives in CT design, and most (85.7%) had informed patients about the results of the trial (Table 4). Notably, all professionals who did not inform the participants about the results declared that they did so because the trial was still ongoing (*not shown*).

**Table 4:** Involvement of CDG professionals and families in CT

	Professionals	Families
	Have you ever been involved (past and present situation) in the development of a CT for CDG? (*n* = 46)	Have you ever participated in a CT for CDG? (*n* = 82)
Yes	30.4%	23.2%
Yes, currently participating in a CT	N.A.	1.2%
No	69.6%	70.7%
I don’t know	N.A.	4.9%

	Professionals	Families
	Have you involved patients and/or patient representatives in the design of the CT? (*n* = 14)	Were you involved in the design of the CT? (*n* = 20)
Yes	64.3%	25.0%
No	35.7%	65.0%
No, and I didn’t know patients/participants/families could be involved in the design of a CT	N.A.	10.0%

	Professionals	Families
	Have you informed the patients who participated in the CT about the results? (*n* = 14)	Were you informed of the results of the CT in which you have participated? (*n* = 20)
Yes	85.7%	35.0%
No, and the trial has already finished	14.3%	25.0%
No, but the trial is still ongoing		35.0%
I don’t know	N.A.	5.0%

CDG families showed high CT awareness (80.5%) (Additional file 8: Figure S8, Panel A). However, their participation was low, with only 24.4% reporting having ever been involved in CTs (Table 4). Among the reasons for not having participated in any CTs, most families (77.4%) said they were not aware of any trials for their CDG (Additional file 11: Table S3). In contrast to the professionals’ experience, only 25.0% of the CDG families reported having been involved in CT design, and 60.0% admitted to not having been informed about CT results, even when they had already ended (25.0%) (Table 4).

**Fig. 5:**
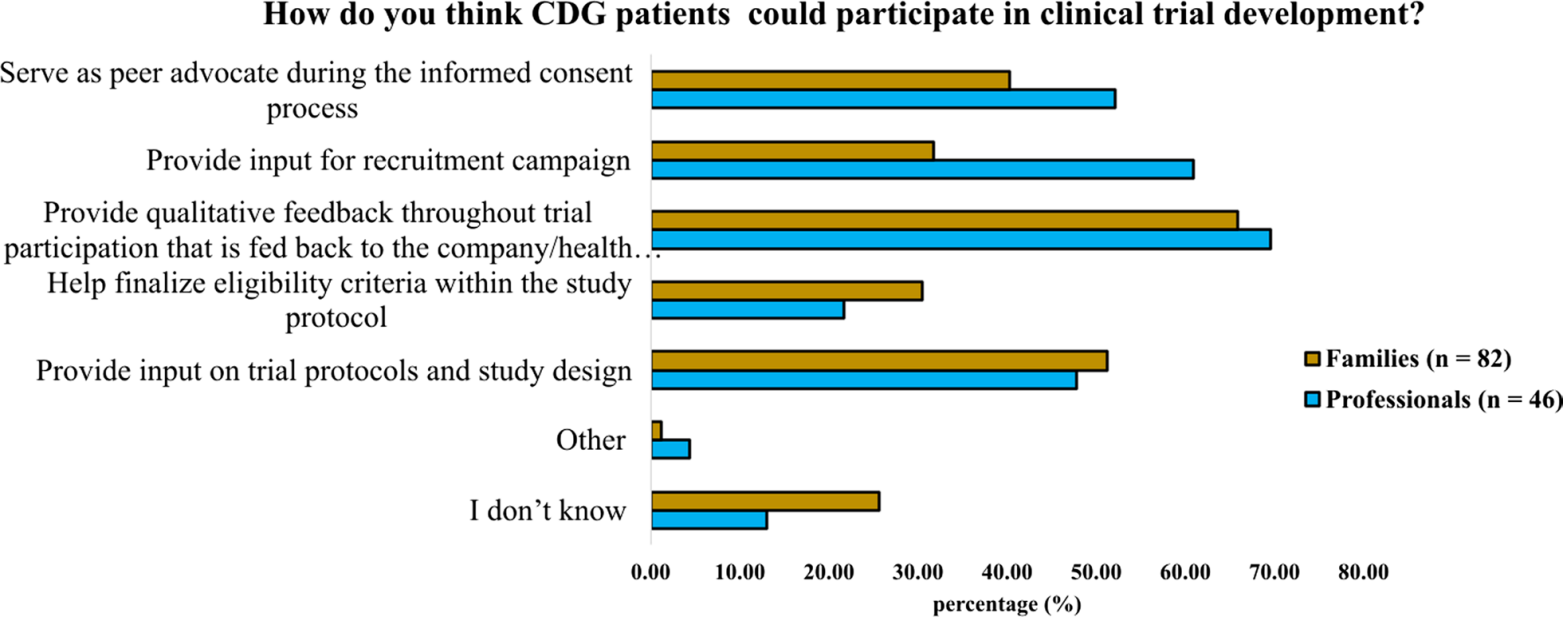
Benefits derived from patients’ involvement in CT development, identified by CDG professionals and families

Among the advantages of family involvement in CT development, the possibility of providing qualitative feedback throughout the trial was acknowledged by the majority of CDG professionals (69.6%) and families (65.8%) (Fig. 5).

Despite their high familiarity with CTs, the CDG community described little experience and participation. Moreover, participants shared opposing perceptions and experiences regarding information-sharing practices and patient/family involvement in CTs.

### CDG community different views, experiences and awareness of doing research “with” patients

CDG patient and family involvement in therapeutic research was highly scrutinised in this study. At times, somewhat contradictory results were shared by the stakeholders. To clarify this point and better explore the CDG community stakeholders’ perspectives on being full and equal research partners, we chose to delve into the CDG community’s views of and experiences with patient-centric research.

Patient (or people) centricity is a research methodology that relies on a co-operational and coeducational model that recognises and captures the differentiated roles (encompassing expertise, needs and priorities) played by all stakeholders—patients, family caregivers and professionals—in improving patients’ health and well-being. Hence, patient-centricity research is developed not “for” patients but “with” patients instead [15–18].

Patient-centric research was not equally perceived or experienced by CDG professionals and families. Professionals were generally aware of what patient-centric research means (71.7% (*not shown*)), and most of them (87.0%) believed that patients should have a voice in research projects (Table 5). While most professionals have experienced patient-centric research, among families, only 41.5% declared having been involved in such a project. Despite their little experience in the field, most CDG families (86.6%) would like to participate in a patient-centric research project (Table 5).

**Table 5:** Views and perceptions of families’ involvement in research projects among CDG professionals and families

	Professionals	Families
	Do you think patients should have a voice in research projects? (*n* = 46)	Given the opportunity, would you be willing to participate in research as a patient/family member? (*n* = 82)
Yes	87.0%	86.6%
No	4.3%	1.2%
I don’t know	8.7%	12.2%

	Professionals	Families
	Have you been involved in any research projects/groups that attempted to incorporate the patient voice? (*n* = 46)	Have you as a patient/family member ever been a research participant? (*n* = 82)
Yes	67.4%	41.5%
No	28.3%	51.2%
I don’t know	4.3%	7.3%

**Fig. 6:**
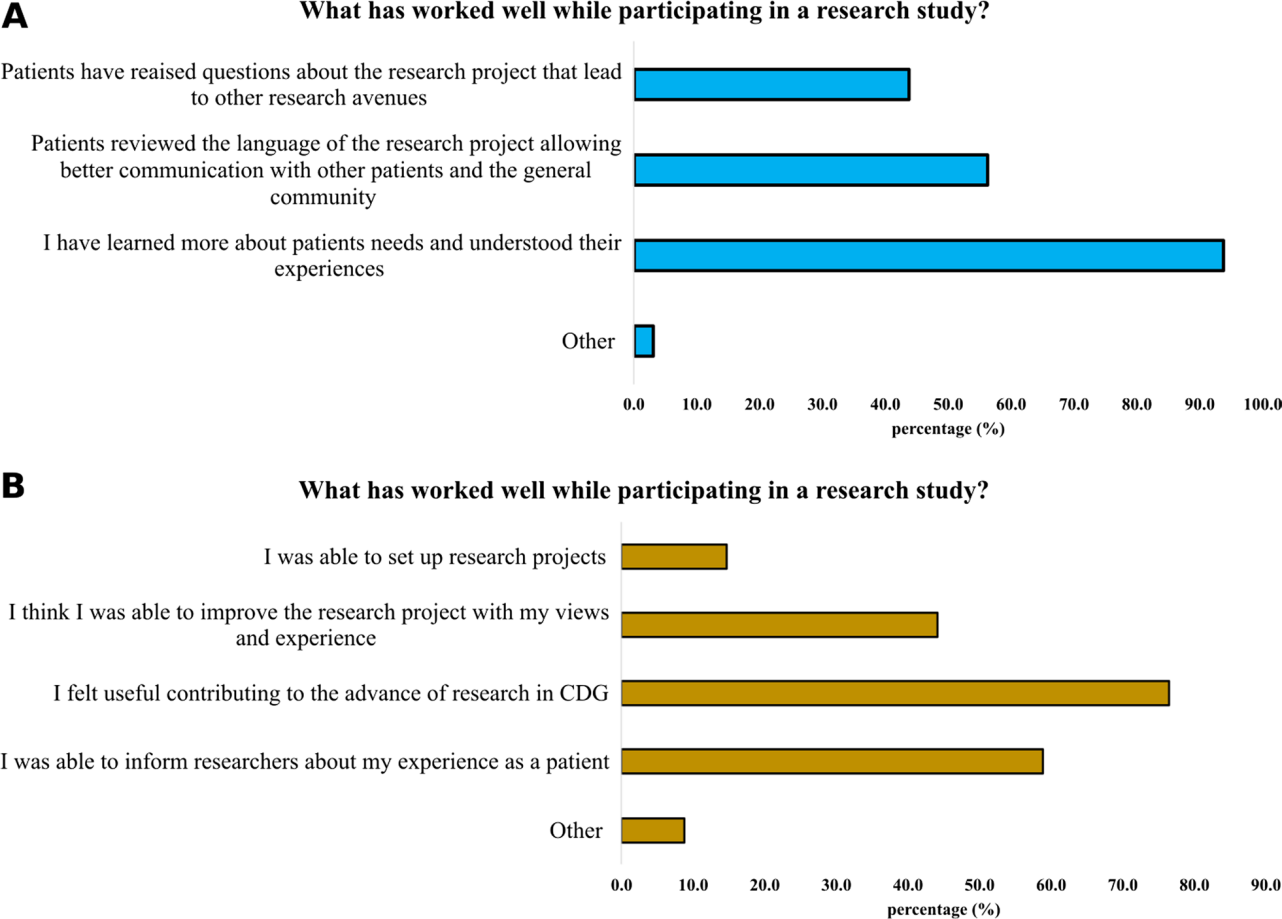
Benefits experienced in patient-centric research studies by CDG professionals (n = 32, **A**) and families (n = 34, **B**)

**Fig. 7:**
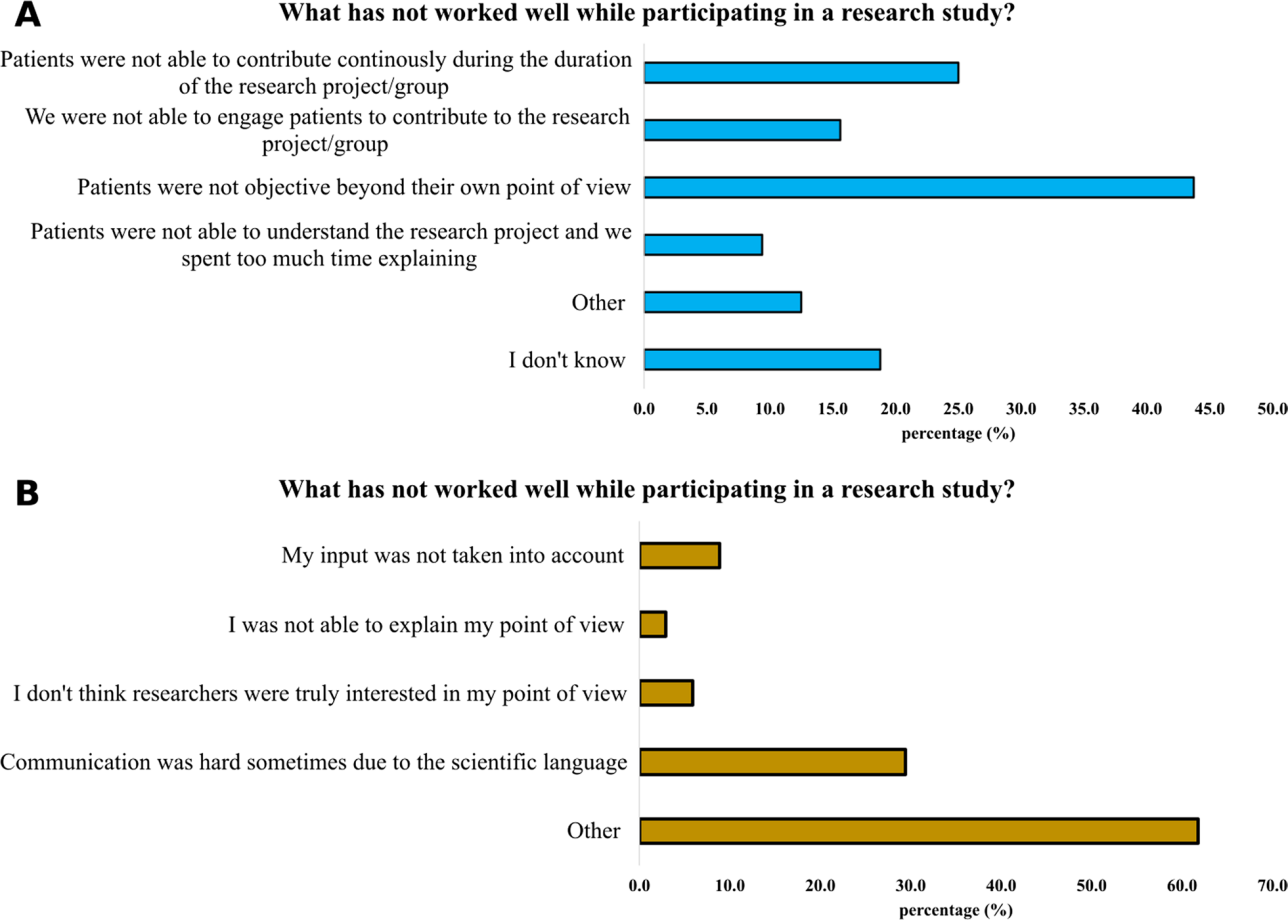
Downsides in patient-centric research by CDG professionals (n = 32, **A**) and families (n = 34, **B**)

Among those professionals (67.4%) and families (41.5%) who had participated in patient-centric research projects, the usefulness of integrating patients’ experiences was recognised (Fig. 6). However, communication challenges—related to scientific language—were identified mainly by families (9.4% of professionals and 29.4% of families). Additional challenges pinpointed by professionals were families’ lack of objectiveness and inconsistent contributions to the project (Fig. 7).

A high percentage of families indicated the “Other” option when asked about the issues faced in participating in a research study, having the opportunity to provide open answers (Fig. 7B). Among them, 28.6% gave positive feedbacks to the experience (for example, “Everything worked well”) and 14.3% stated the answer was not applicable. 19.0% was not satisfied in the feedbacks received, while the remaining ones described their personal experience, highlighting travel costs problems, slowness of the research and absence of people-centric research projects for their CDG.

Patient-reported outcomes measures (PROMs) are any type of reporting that describes patients' perspective about the impact of their condition or its treatment. They are subjective measures of how a patient feels or functions due to a disease or any intervention. PROMs have increasingly been used in drug development to integrate the patient experience as an informative and/or decisive clinical outcome, supporting drug approval and informing labelling claims. Consequently, PROMs are an essential and effective tool to boost patient-centric research.

PROMs awareness among professionals, according to our survey, was 47.8% (; however, it was still remarkably higher when compared to that of CDG families. Indeed, almost 80.0% of family participants did not know what PROMs are (Additional file 8: Figure S8, Panel D).

CDG professionals reported greater familiarity and experience with patient-centric research and PROMs. Nonetheless, families showed high availability for and interest in participating in patient-centric research. Both stakeholder groups recognised the value of integrating the patient voice, although they also identified that, to effectively do so, communication challenges must be overcome.

### Artificial intelligence as a therapeutic discovery accelerator

Therapeutic discovery relies on new generation tools and technologies, and this dependency is predicted to continue increasing. Artificial intelligence (AI), mainly through machine learning (ML, a subtype of AI), provides algorithms capable of learning from data. According to the USA Food and Drug Administration, AI is “the science and engineering of making intelligent machines”, while ML is “an AI tool that can be used to design and train software algorithms to learn from and act on data” [11, 19]. AI has diverse applications in therapeutic research, namely in drug discovery and preclinical research (e.g., drug repositioning, molecules design and interaction, reduction of animal testing) and even in CT design (e.g., patients’ identification and recruitment, and discovery of new disease biomarkers) [11]. Hence, we sought to investigate the opinions and experiences of CDG stakeholders with AI.

Although CDG professionals described limited AI use in their research (23.9%) (Additional file 2: Figure S2, Panel D), the majority (67.4%) agreed with its helpfulness in finding new therapies (*not shown*). AI was also perceived as a therapy accelerator by most families (54.0%), who interestingly showed high AI awareness levels (76.8%) (Additional file 5: Figure S5, Panel B).

**Fig. 8:**
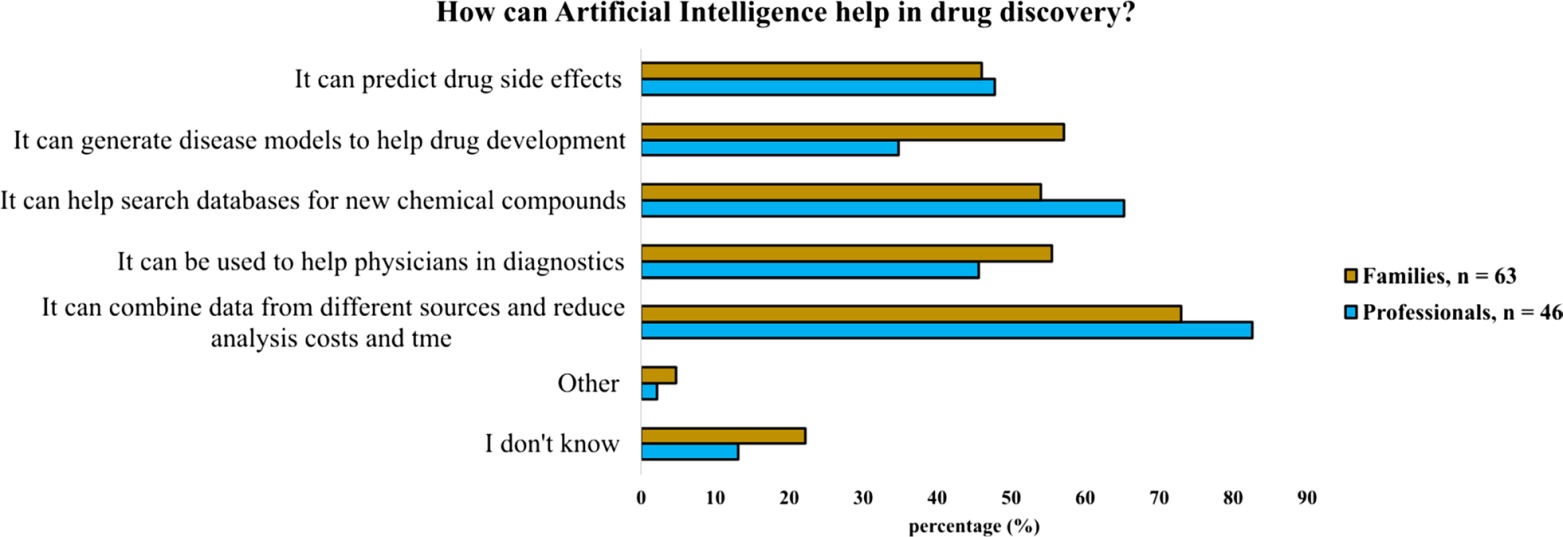
Potential applications of AI to CDG drug development according to professionals and families

Regarding the CDG stakeholders’ views of the roles that AI could play in advancing drug discovery, professionals and families generally expressed convergent opinions, especially about the possibility of AI enabling the combination of different sources of data, thus reducing the time and costs of the analysis (82.6% of professionals and 73.0% of families). Additionally, professionals and families identified AI as a clinical decision support tool for clinicians, namely for diagnosis and drug side effect prediction. Despite this agreement, not all topics were equally perceived by the stakeholders. Thus, professionals were more confident about AI’s supportive role in searching databases for new chemical compounds (65.2%), while families expressed their trust in AI as a booster for the generation of new disease models (57.1%) (Fig. 8).

The CDG community collectively displayed a positive vision of AI’s multipurpose role in drug discovery and development, seeing it as a relevant clinical support decision tool. Families also reported high AI awareness, which was in contrast with professionals' current limited AI use in their CDG research.

## Discussion

Drug development and approval are multistep processes that require the involvement and expertise of various stakeholders, including professionals and family members.

The present research collected and analysed the awareness levels, views and experiences of CDG professional and family stakeholders with the drug development and approval processes. This community-centric and comparative work created a comprehensive overview of the CDG community drug development landscape, mapping gaps and opportunities to improve therapeutic research for CDG. Importantly, capturing the CDG professionals’ and families’ perspectives and identifying both common and stakeholder-specific needs and expectations could guide the development of general and tailored activities.

We identified several educational, communication and research challenges and opportunities. Regarding educational needs, the identified varying familiarity levels reported by CDG professionals and families about the different drug development and approval tools explored in this work highlight the need to offer information and training on these topics. Families indicated a lack of CDG awareness much more than professionals, likely because of their everyday experience. Some initiatives dedicated to empowering the community about these subjects have been flourishing. Notably, the World Organisation platform is creating content dedicated to drug development emphasising CTs (https://worldcdg.org/index.php/clinical-trials/explore-clinical-trials; https://worldcdg.org/index.php/clinical-trials/understanding-clinical-trials). Additionally, CDG CARE, a relevant CDG patient organisation, has developed several webinars dedicated to the “ABCs of clinical trials” (https://cdgcare.org/abcs-of-clinical-trials/).

Various results have indicated inefficient communication between CDG professionals and families regarding communication gaps. The most striking findings are the conflicting perceptions regarding patient communication about CTs. In particular, most families admitted to not having been informed about CT results, in contrast with professionals’ statements. This finding is in line with a recent study pointing to a gap in the timely and effective communication of CT results to patients and the general public; only 2.0% of all CT returned a plain language summary of the study results to participants in 2018 [20]. The percentage of families who did not know whether their clinical data had ever been collected for a patient registry further underscores these challenges. The return of plain-language results summaries, among other patient engagement initiatives, improves CT recruitment and retention rates. However, the low incentive to provide plain language summaries is perceived by research sponsors as the most significant barrier [20].

Several research-related challenges and opportunities were also mapped. Most of CDG families were not aware of CTs for their CDGs, clearly demonstrating different advancement levels among different CDGs. In fact, therapeutic research initiatives favour PMM2-CDG [10, 21, 22]. This discrepancy could be perceived as an isolating tendency towards the different CDG, thus not helping families’ involvement. Despite this discrepancy, NHS including several CDGs (NCT04199000) and research studies targeting the same therapeutic strategy for different CDGs (e.g., galactose supplementation) are a reality (https://worldcdg.org/drug-development/pipeline). Furthermore, learning from preclinical and clinical research, and the same therapeutic strategies, can be applied to different CDGs.

Conflicting results were also observed regarding patient-centric research awareness and experience. Less than 50.0% of the families reported being involved in a patient-centric research project, contrasting with the professionals’ views; most of these professionals reported being involved in patient-centric research projects. Patient-centric research must be implemented and accepted at all levels of an organisation in both the public and private sectors [23]. Hence, the different experiences among families and professionals could stem from different notions of patient involvement among the stakeholders. Patients and their families have been solicited for inclusion in all decisions about research on rare diseases, including involvement in the decision-making processes of research collaborations and networks [24]. However, this involvement might imply a high level of commitment and time that might not be possible for all patients and/or families due to the disease burden. High participation levels have largely demonstrated the pro-research attitude of the CDG families in several patient-centric research studies [12–14]. This fact, and the value of patient participation demonstrated by both stakeholders indicates a strong willingness to cooperate.

Other research challenges are limited access and use of therapeutic research tools, such as biobanks and disease models [25]. However, they are not commonly used by most CDG researchers. The type of biobank (e.g., single research team collections or large networks) and the nature of the biological material stored can influence the relevance of biobanks for CDG researchers [25]. Integrating omics data in biobanks and the combination of biobanks and patient registries could improve biobank usefulness in rare diseases, such as CDGs, thus increasing their use [25, 26]. Furthermore, the absence of sample centralisation affects sample sharing, hindering CDG therapeutic research. The lack of funding was identified by CDG professionals as a major pitfall for CDG research and ultimately therapy development. Public funding still represents the major funding source for CDG research. The lack of national initiatives to promote rare disease research and the difficulty in finding and securing funding for basic and translational research represent hurdles for drug development [27, 28]. In addition, the lack of collaboration between researchers and pharmaceutical companies was also pointed out by CDG researchers as an obstacle. The cooperation between pharmaceutical companies, researchers and patients through public–private partnerships (PPPs) could help to overcome this challenge [29, 30]. In contrast, families did not perceive the lack of collaboration between researchers and pharmaceutical companies.

AI has boosted both diagnosis and classification as therapeutic developments in rare diseases [11]. In CDG, AI has been used to elucidate basic disease mechanisms and facilitate diagnosis, classification and characterisation. AI tools for therapy discovery in CDG have been limited, indicating that this research area is promising [11, 31]. Consequently, a shared opinion among stakeholders is that AI tools would lead to time and cost reductions in drug development. Creating greater AI know-how among the professional community should be a priority to boost therapeutic development for CDG.

We aware that this research may have some limitations. The first is that there was a language barrier since the surveys were only made available in English, which could have limited the participation of non-English speakers. In previous e-surveys provided in different languages, the participation level was superior, particularly when comparing the length of the recruitment period (e.g., the e-questionnaire for liver assessment in CDG—LeQCDG, which was available for 42 days and obtained 155 replies), despite the English version being the most represented [13, 14]. Second, regarding recruitment bias, and in addition to distribution on social media channels, the survey was distributed by email to registered participants of the 4th edition of the World Conference on CDG, which might have increased participation from conference participants. Besides this, only 36.0% (n = 46) of the 128 study participants were professionals, and of those, 50.0% were researchers. This finding could be due to recruitment bias since most professionals attending the 4th World CDG Conference were researchers and/or clinicians involved in research. Finally, unfortunately in some of the survey sections (e.g., disease models and biobanks), the questions for patients and professionals varied, limiting direct comparison between the answers and, consequently the data analysis. However, these differences were also planned to accommodate the different knowledge levels foreseen between the families and professionals regarding the drug development process.

Despite these limitations, we believe that our study has major strengths. Firstly, we used an innovative and patient-inclusive methodology that allowed us to capture the needs of the CDG community and identify knowledge gaps that could help to tailor information materials and empowerment strategies. Surveys are a valuable source of data and their use is increasing in rare diseases [32–34], allowing us to overcome the geographically dispersed nature of these communities, as the CDG community. Benefits related to e-questionnaires help bring researchers closer to patients and boost people-centric research. Many aspects can be targeted, spacing from general to very symptom-specific aspects [35–45]. The Rare Barometer project by EURORDIS is a successful example which aims to gather worldwide data to boost knowledge across rare diseases [46]. With the present approach, using an online platform to administer the survey, we could reach and capture the needs and views of a larger audience. Secondly, the identification of the community’s needs and preferences helped to shape the agenda of the 4th World Conference on CDG, which was highly relevant to provide the families with information on the topics that were most urgent for them and ensure that their information needs were met, increasing engagement and participation. Finally, the inclusion of a piloting phase with members of both the professional and family communities ensured the refinement and/or elimination of content and understandability issues and ensured patient engagement and participation from the project’s beginning.

Experiences deriving from other rare diseases communities can represent a major opportunity for patient-centricity and research. Recently, Ayayj et al. developed a centralized clinical data repository for the Dup15 syndrome involving researchers, physicians and families [47]. Their project included the collection of clinical data and survey information, through the involvement of the stakeholders. A similar database has been built by Petrossians et al., called the Liege Acromegaly Survey (LAS) [48]. The LAS is presented as a new relational database to be used for clinical research and allowing to strengthen statistical analyses by pooling anonymous patient data. Also, Johnson et al. developed a completely web-based patient registry, that allowed the easy involvement of many patients in a brief time [49]. Interestingly, almost all the participants (94%) indicated willingness for providing biological samples. This is a very interesting approach to overcome the barrier of lack of samples highlighted by the CDG Community.

Benefits deriving from the cooperation among different communities would strengthen patient-centricity and boost research. This is a major point in rare diseases field, as it is the necessity of gathering the information collected in the different registries databases [50].

## Conclusions

Our study assessed the CDG community perspectives and experience with drug development and approval using an innovative and inclusive methodology. Including the CDG professionals’ and families' voices resulted in more integrative comprehension of the CDG drug development landscape.

The evidence from this study suggests various educational and informational gaps about several aspects of drug development among families. These gaps can be addressed with targeted and effective educational campaigns and materials to boost education among the CDG community. Communication needs and inconsistencies were signalled by both stakeholder groups, emphasising the urgency to improve communication strategies (e.g., languages and messages) to ensure proper research participation and inclusion. Despite the limited experience of the CDG families and professionals with the overall drug development process, families demonstrated a high pro-research attitude, with both stakeholders valuing the role of patients in research and patient-centric research projects. This finding points to the need for further fostering and strengthening patient-centric research projects that promote active patient participation to advance drug development for CDG.

## Supplementary Information

**Additional file 1: Figure S1.** Example of the survey recruitment posts.

**Additional file 2: Figure S2.** CDG professionals’ experiences in pre-clinical research

**Additional file 3: Figure S3.** Major difficulties with drug development identified by CDG professionals

**Additional file 4: Figure S4.** Funding sources for CDG research identified by professionals

**Additional file 5: Figure S5.** CDG families’ awareness about pre-clinical research tools

**Additional file 6: Figure S6.** Major obstacles to implementing CDG biobanks identified by CDG professionals

**Additional file 7: Figure S7.** Commonly used models for CDG therapeutic research, identified by professionals

**Additional file 8: Figure S8**. CDG families’ awareness about clinical research tools

**Additional file 9: Table S1.** E-survey participants’ geographical distribution

**Additional file 10: Table S2.** CDG patients’ roles in the drug approval process, identified by professionals

**Additional file 11: Table S3.** Major obstacles to participation in clinical trials, identified by CDG families

**Additional file 12.** E-survey - version adapted to professionals.

**Additional file 13.** E-survey - version adapted to families.

## Data Availability

Data supporting the conclusions in this article are included within the article itself. Further data is available on request.
