# Supplementary figures and images for: Stakeholders’ views on drug development: the congenital disorders of glycosylation community perspective
Source: Orphanet J Rare Dis. 2022 Jul 30;17:303. doi: 10.1186/s13023-022-02460-0 (PMC9338569; doi:10.1186/s13023-022-02460-0)

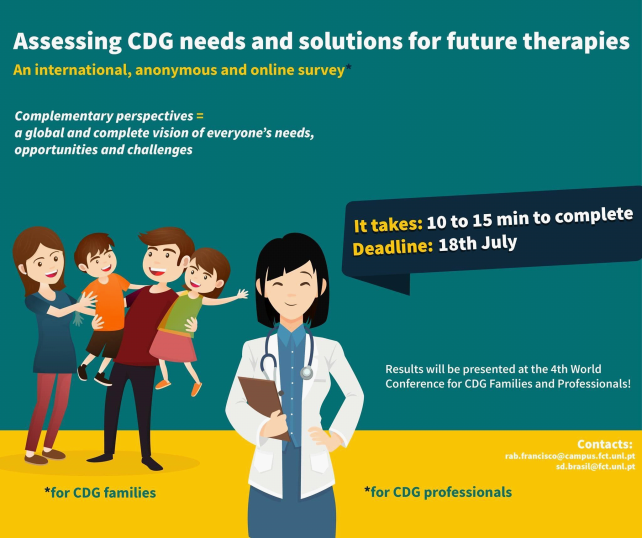

Supplement: Supplementary file 1 — Additional file 1: Figure S1. Example of the survey recruitment posts. [file 13023_2022_2460_MOESM1_ESM.png]

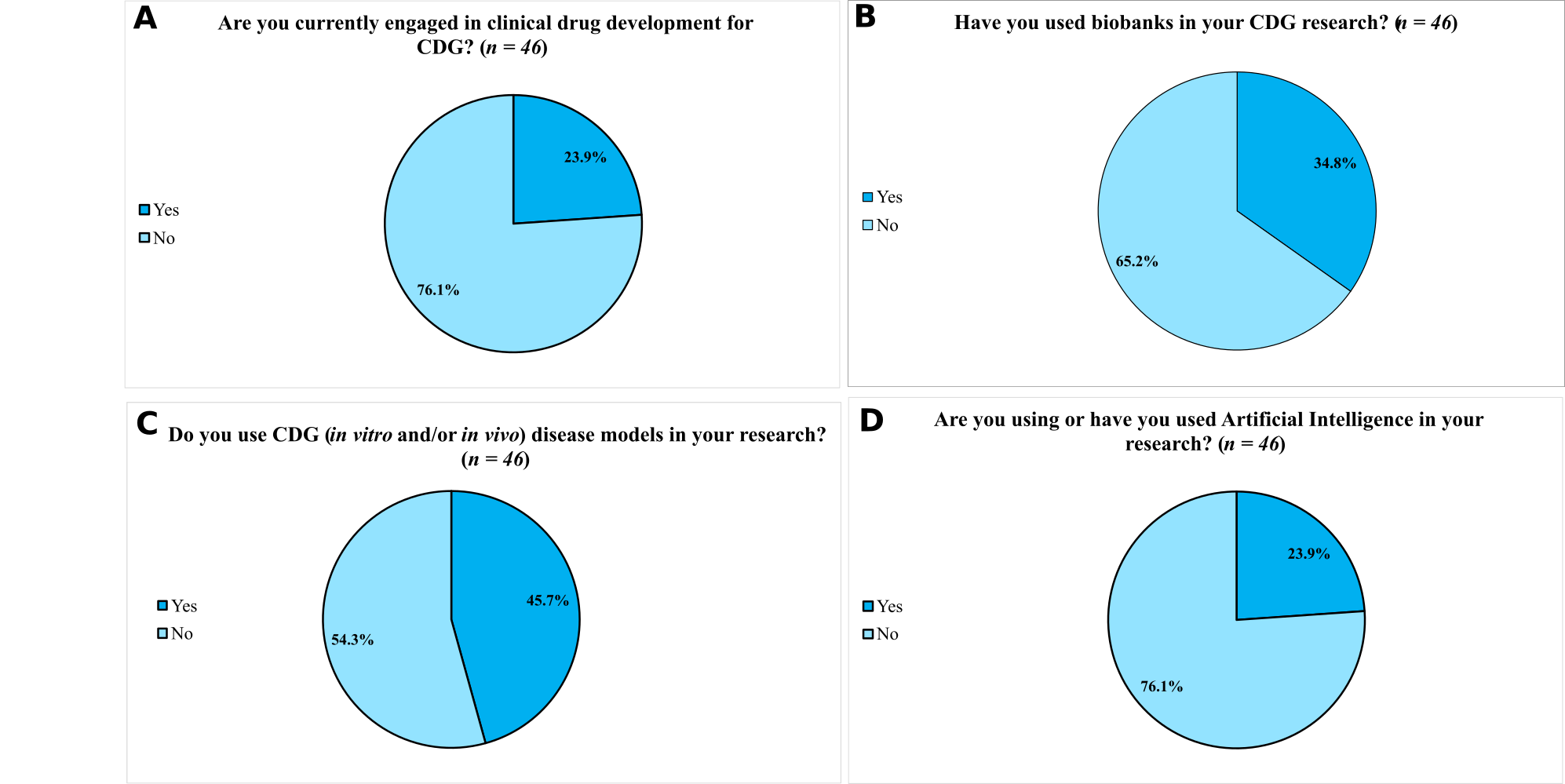

Supplement: Supplementary file 2 — Additional file 2: Figure S2. CDG professionals’ experiences in pre-clinical research [file 13023_2022_2460_MOESM2_ESM.png]

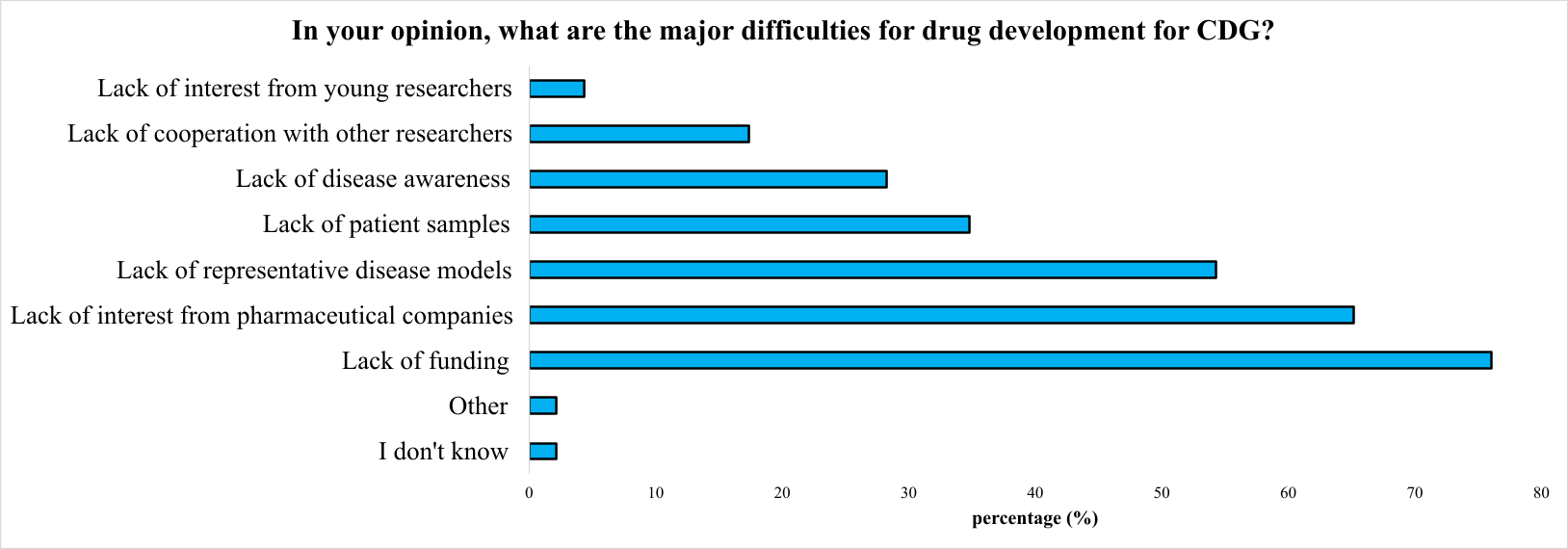

Supplement: Supplementary file 3 — Additional file 3: Figure S3. Major difficulties with drug development identified by CDG professionals [file 13023_2022_2460_MOESM3_ESM.png]

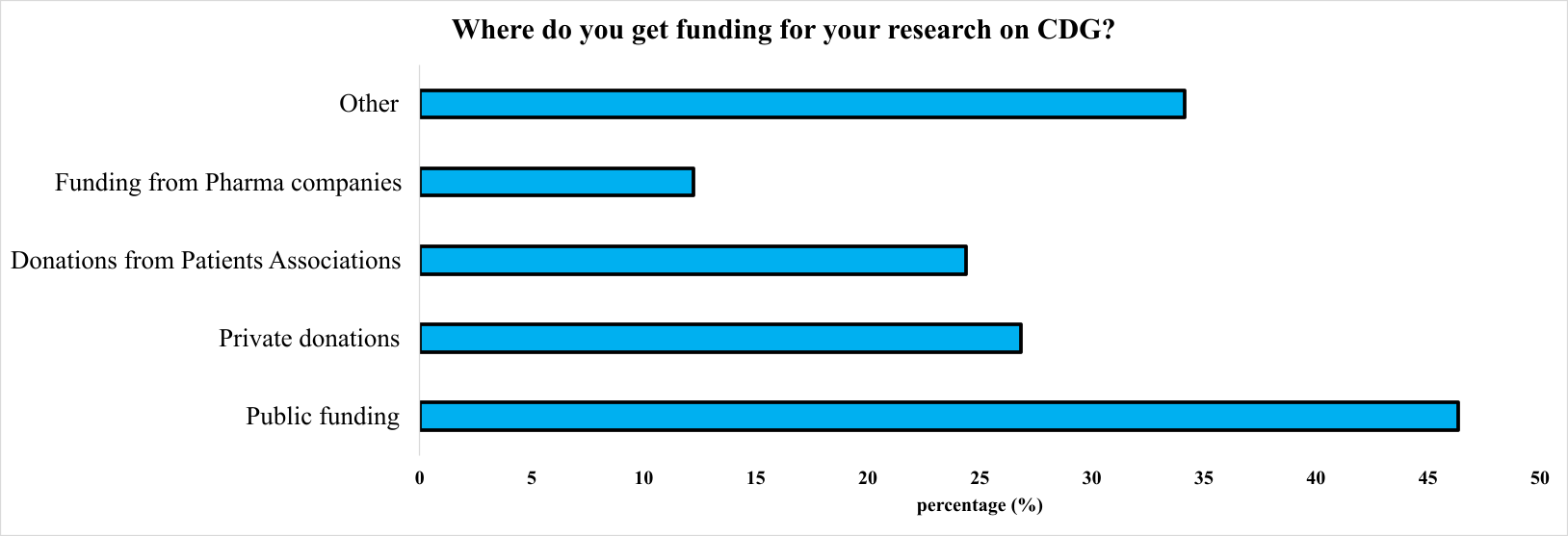

Supplement: Supplementary file 4 — Additional file 4: Figure S4. Funding sources for CDG research identified by professionals [file 13023_2022_2460_MOESM4_ESM.png]

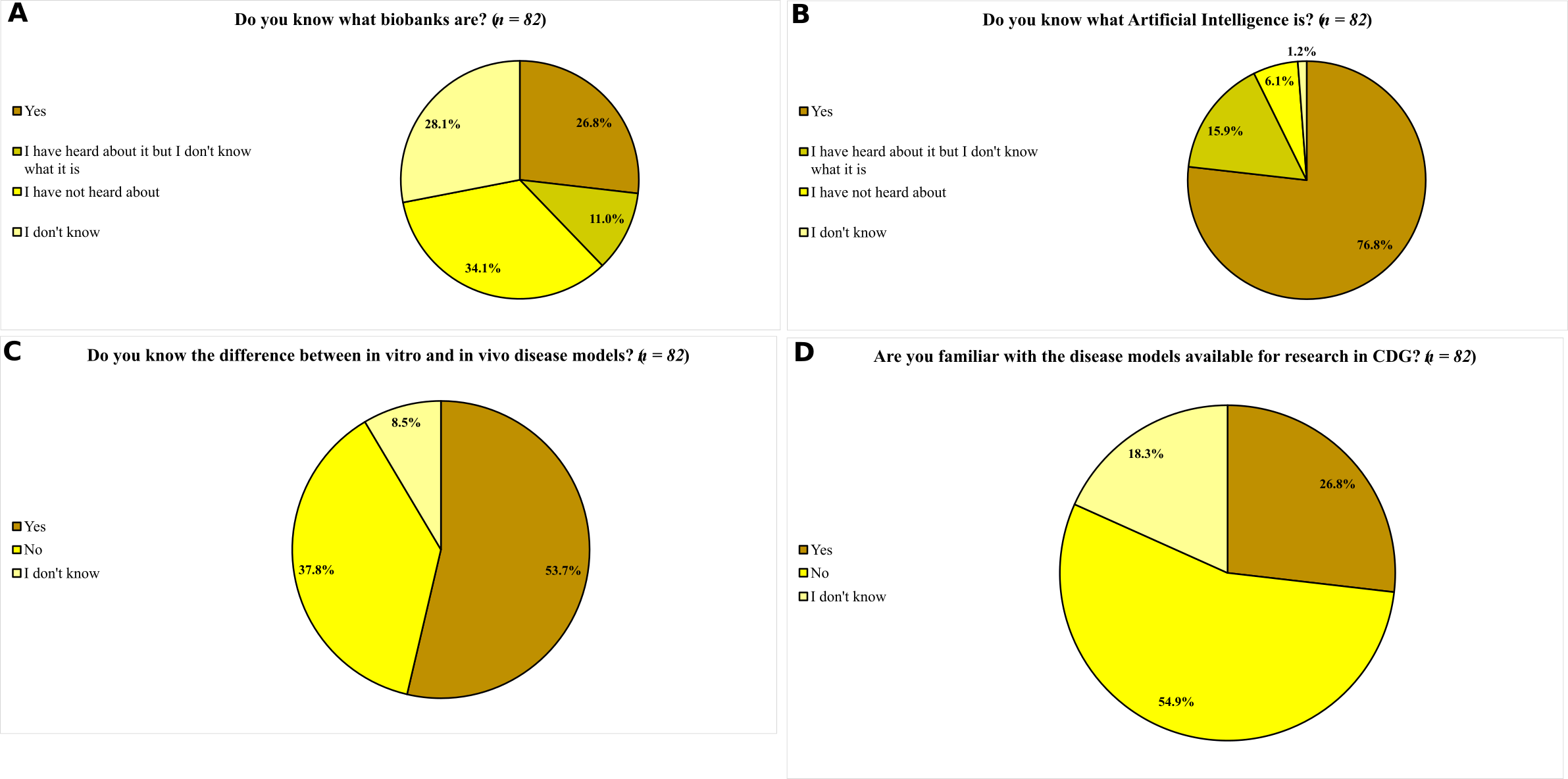

Supplement: Supplementary file 5 — Additional file 5: Figure S5. CDG families’ awareness about pre-clinical research tools [file 13023_2022_2460_MOESM5_ESM.png]

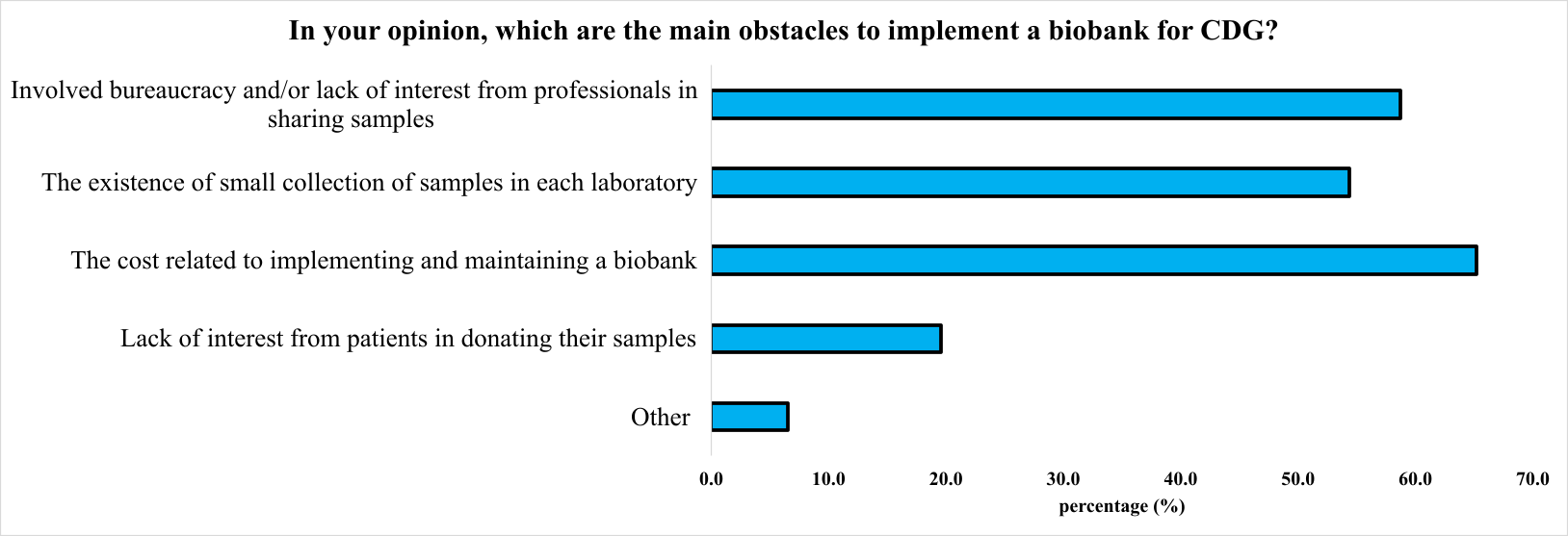

Supplement: Supplementary file 6 — Additional file 6: Figure S6. Major obstacles to implementing CDG biobanks identified by CDG professionals [file 13023_2022_2460_MOESM6_ESM.png]

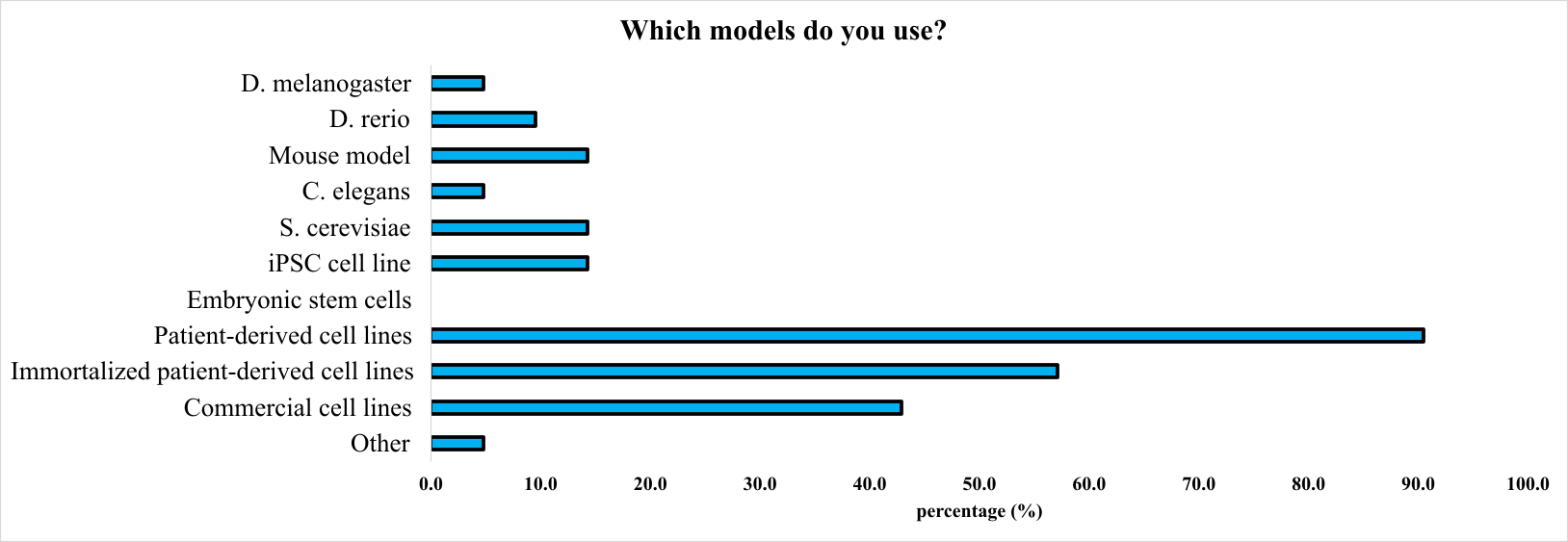

Supplement: Supplementary file 7 — Additional file 7: Figure S7. Commonly used models for CDG therapeutic research, identified by professionals [file 13023_2022_2460_MOESM7_ESM.png]

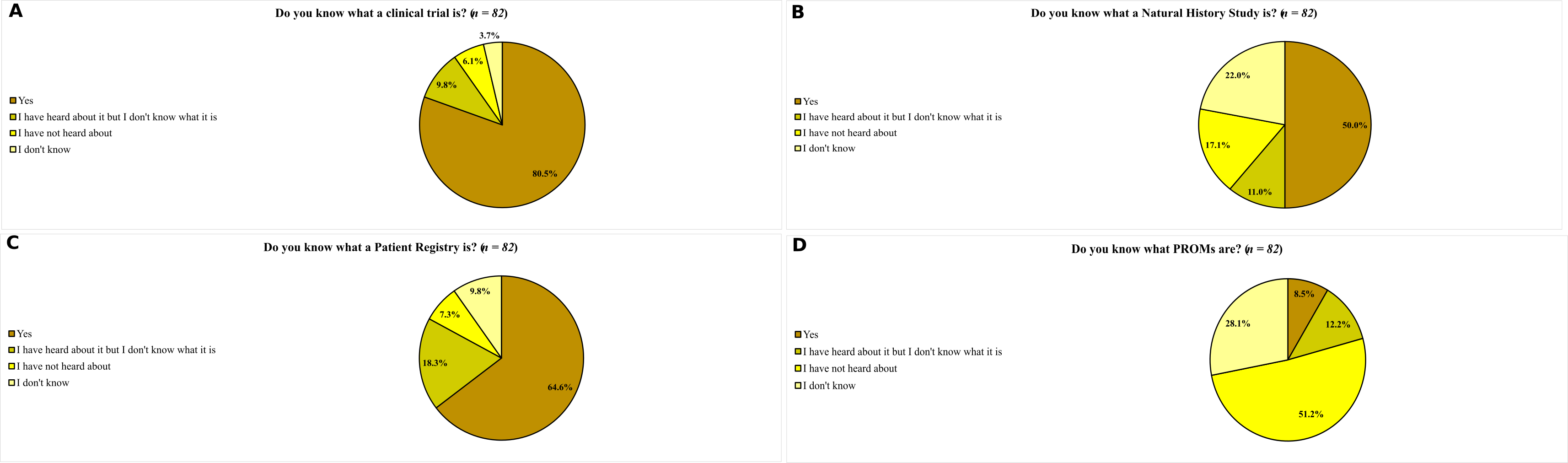

Supplement: Supplementary file 8 — Additional file 8: Figure S8. CDG families’ awareness about clinical research tools [file 13023_2022_2460_MOESM8_ESM.png]
