# Supplementary material for: Stakeholders’ views on drug development: the congenital disorders of glycosylation community perspective
Source: Orphanet J Rare Dis. 2022 Jul 30;17:303. doi: 10.1186/s13023-022-02460-0 (PMC9338569; doi:10.1186/s13023-022-02460-0)
Supplement: Supplementary file 9 — Additional file 9: Table S1. E-survey participants’ geographical distribution [file 13023_2022_2460_MOESM9_ESM.docx]

**Supplementary table 1:** E-survey participants’ geographical distribution.

| **In which country do you live?** | | |
| --- | --- | --- |
|  | **PROFESSIONALS** (*n = 46*) | **FAMILIES** (*n = 82*) |
| Armenia | - | 1.2% |
| Australia | 2.2% | 8.5% |
| Austria | - | 2.4% |
| Belgium | 4.3% | 1.2% |
| Brazil | 2.2% | 1.2% |
| Bulgaria | 2.2% | - |
| Canada | - | 2.4% |
| Czech Republic | 6.5% | 1.2% |
| Denmark | - | 1.2% |
| Estonia | - | 2.4% |
| France | 4.3% | 4.9% |
| Georgia | - | 1.2% |
| Germany | - | 1.2% |
| Iran (Islamic Republic of) | 2.2% | - |
| Italy | 8.7% | 2.4% |
| Lithuania | 2.2% | - |
| Netherlands | 2.2% | 6.1% |
| Norway | 2.2% | - |
| Portugal | 19.6% | 6.1% |
| Romania | - | 1.2% |
| Russian Federation | - | 2.4% |
| Slovakia | 2.2% | - |
| Slovenia | - | 1.2% |
| South Africa | 2.2% | 3.7% |
| Spain | 15.2% | 7.3% |
| Sweden | 2.2% | 3.7% |
| Switzerland | - | 1.2% |
| United Arab Emirates | - | 1.2% |
| United Kingdom of Great Britain and Northern Ireland | 4.3% | 2.4% |
| United States of America | 15.2% | 31.7% |
