# Supplementary material for: Stakeholders’ views on drug development: the congenital disorders of glycosylation community perspective
Source: Orphanet J Rare Dis. 2022 Jul 30;17:303. doi: 10.1186/s13023-022-02460-0 (PMC9338569; doi:10.1186/s13023-022-02460-0)
Supplement: Supplementary file 10 — Additional file 10: Table S2. CDG patients’ roles in the drug approval process, identified by professionals [file 13023_2022_2460_MOESM10_ESM.docx]

**Supplementary table 2:** CDG patients’ roles in the drug approval process, identified by professionals.

| PROFESSIONALS | |
| --- | --- |
| In your opinion, how can CDG patients participate in the drug approval process? *(n = 31)* | |
| Preparation of risk management plan summaries during the evaluation process | 35.5% |
| Participation as members of EMA committees | 61.3% |
| Increasing disease awareness | 87.1% |
| Lobbying for approval | 80.6% |
| Other | 6.4% |
