# Supplementary material for: Stakeholders’ views on drug development: the congenital disorders of glycosylation community perspective
Source: Orphanet J Rare Dis. 2022 Jul 30;17:303. doi: 10.1186/s13023-022-02460-0 (PMC9338569; doi:10.1186/s13023-022-02460-0)
Supplement: Supplementary file 11 — Additional file 11: Table S3. Major obstacles to participation in clinical trials, identified by CDG families [file 13023_2022_2460_MOESM11_ESM.docx]

**Supplementary table 3:** Major obstacles to participation in clinical trials, identified by CDG families.

| FAMILIES | |
| --- | --- |
|  | **If applicable, why haven’t you participated in a clinical trial?** *(n = 62)* |
| I was not aware of any clinical trial for my CDG type | 77.4% |
| I did not fit the inclusion criteria of the study | 9.7% |
| The clinical trial site is too far from my hometown | 9.7% |
| The clinical trial site is too far from my home and I do not have financial possibilities to go there | 6.4% |
| The medical insurance did not cover the expenses | 1.6% |
| The disease has stabilized and I don’t think a clinical trial would be useful | 4.8% |
| Other | 25.8% |
