# Supplementary material for: Stakeholders’ views on drug development: the congenital disorders of glycosylation community perspective
Source: Orphanet J Rare Dis. 2022 Jul 30;17:303. doi: 10.1186/s13023-022-02460-0 (PMC9338569; doi:10.1186/s13023-022-02460-0)
Supplement: Supplementary file 12 — Additional file 12. E-survey - version adapted to professionals. [file 13023_2022_2460_MOESM12_ESM.pdf]

4th World Conference CDG Questionnaire for Professionals – Assessing CDG needs and solutions for future therapies.

4th World Conference CDG Questionnaire - Instructions and informed consent for participants

4th World Conference CDG Questionnaire – Assessing CDG needs and options for the future

Before deciding or not to participate, it’s important that you understand the purpose of this study.
By agreeing to participate, you are agreeing with the terms and conditions explained in this document.

About the questionnaire
The full comprehension of the therapy landscape for CDG requires the consultation and involvement of all stakeholders. Also, it is imperative to educate and empower the CDG Patient Community, besides promoting interaction and communication among all members of the community. Researchers, medical professionals and representatives of the pharmaceutical industry play a pivotal role in this education and communication process. Hence, this questionnaire intends to assess the level of knowledge and awareness about various aspects related to the drug development process among the Congenital Disorders of Glycosylation (CDG) research community (researchers, clinicians, industry representatives, etc.).

The main goals of this questionnaire are to:

- Pool knowledge on CDG therapy research and drug development process from all the CDG community stakeholders;
- Identify the knowledge gaps and needs among the CDG Community;
- Evaluate and compare the perspectives of CDG Patients/Caregivers vs Professionals;
- Identify the possible common solutions to increase knowledge regarding drug development process.

The Team
This project has been developed by:

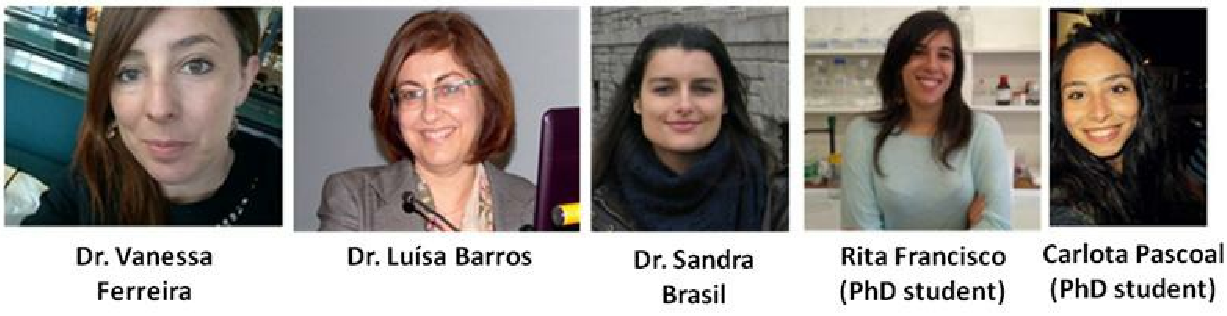

This **questionnaire** will be available **only in English** and should **take about 15 – 20 minutes to complete**.

NOTE THAT you don't have to fill in the entire questionnaire all at once. You can stop at any point, how many times you need and finish it when it's convenient to you as long as it is done within the deadline of the project. To ensure that you can return to the question you were at when you left the survey, MAKE SURE you use the SAME DEVICE (e.g. computer or phone) and INTERNET BROWSER (e.g. Google Chrome).

Who can participate?
You can participate if you are a researcher, clinician, clinician involved in research, industry representative (in any of the cases you need to be 18 years old or older).
Participation is voluntary.
At any stage of the questionnaire you can stop filling it out. You don't need to justify your decision and that won't affect you in anyway.

Will my information be published?
This survey is anonymous. We only ask for the necessary information to analyze the results. No personal information will be published. Data will be analysed and the results of this survey will be presented at the **4th World Conference for CDG** and used for further scientific publications that will be made available on APCDG (www.apcdg.com) and CDG & Allies – PPAIN (www.researchcdg.com) websites.

Personal data collection abides the Directive (EU) 2016/679 from 27th April 2016.

Who can I contact if I have doubts?
If you need additional information about the questionnaire, language or clarifications on the content, please do not hesitate to write to the following address: sd.brasil@fct.unl.pt

* 1. **ELECTRONIC CONSENT:** Please select your choice below.
Clicking on the "agree" button below indicates that:

· **You have read and understood the previous information,**
· **You voluntarily agree to participate,**
· **You are a medical student, a general practitioner or other medical specialty.**

If you wish to participate in the research study, please accept participation by clicking on the "agree" button.

- ◯ Agree
- ◯ Disagree

* 2. Are you a...

- ◯ Researcher
- ◯ Clinician
- ◯ Clinician involved in research
- ◯ Company representative
- ◯ Other (please specify)

* 3. In which country do you live in?

Workshop 1 – Research and development

* 4. Do you know what patient-centric research means?

- ◯ Yes
- ◯ I have heard about patient-centric research but I don’t know what it is
- ◯ I have not heard about patient-centric research
- ◯ I don't know

* 5. Do you think patients should have a voice in research projects?

- ◯ Yes
- ◯ No
- ◯ I don't know

* 6. Have you been involved in any research projects/groups that attempted to incorporate the patient voice?

- ◯ Yes
- ◯ No
- ◯ I don't know

* 7. What do you think has worked well (please select ALL that apply)?

- [ ] I have learned more about patients needs and understood their experiences
- [ ] Patients reviewed the language of the research project allowing better communication with other patients and the general community
- [ ] Patients have raised questions about the research project that lead to other research avenues
- [ ] Other (please specify)

* 8. What do you think has not worked well (please select ALL that apply)?

- [ ] Patients were not able to understand the research project and we spent too much time explaining
- [ ] Patients were not objective beyond their own point of view
- [ ] We were not able to engage patients to contribute to the research project/group
- [ ] Patients were not able to contribute continuously during the duration of the research project/group
- [ ] I don't know
- [ ] Other (please specify)

* 9. How important are biobanks for CDG therapy-driven research?

Please note that:
0 = I don't think biobanks are important for CDG therapy-driven research.
5 = I think biobanks are essential for CDG therapy-driven research.

0	1	2	3	4	5
○	○	○	○	○	○

* 10. Have you used biobanks in your CDG research?

○ Yes

○ No

* 11. Which biobank(s) do you use?

* 12. Why not (please select ALL that apply)?

- [ ] I don’t know any biobanks for CDG
- [ ] Biobanks don’t apply to my research
- [ ] Never felt the need to use them
- [ ] Other (please specify)

* 13. Which are the main obstacles to implement a biobank for CDG (please select ALL that apply)?

- [ ] Lack of interest from patients in donating their samples
- [ ] The cost related to implementing and maintaining a biobank
- [ ] The existence of small collection of samples in each laboratory
- [ ] Involved bureaucracy and/or lack of interest from professionals in sharing samples
- [ ] Other (please specify)

Workshop 2 – Tools for preclinical and clinical drug development and approval

* 14. Are you familiar with the clinical drug development and approval process?

- ○ Very familiar
- ○ Familiar
- ○ Slightly familiar
- ○ Not familiar
- ○ I don't know

* 15. Are you currently engaged in clinical drug development for CDG?

- ○ Yes
- ○ No

* 16. What are the major difficulties for drug development for CDG (please select ALL that apply)?

- ☐ Lack of funding
- ☐ Lack of cooperation with other researchers
- ☐ Lack of representative disease models
- ☐ Lack of disease awareness
- ☐ Lack of patient samples
- ☐ Lack of interest from pharmaceutical companies
- ☐ Lack of interest from young researchers
- ☐ I don't know
- ☐ Other (please specify)

* 17. Are you or have you been involved in (e.g. set up or contribute with data) a patient registry for CDG?

- ◯ Yes
- ◯ No
- ◯ No, and to the best of my knowledge there are no patient registries for CDG

* 18. What challenges did you find (please select ALL that apply)?

- ☐ Difficulty in patient recruitment
- ☐ Difficulty in setting relevant endpoints
- ☐ Difficulty in defining which information should be collected
- ☐ High number of CDG
- ☐ Elevated costs related to implementation and maintenance
- ☐ Missing data points along the time
- ☐ Other (please specify)

* 19. Have you ever been involved in a Natural History Study (NHS) for CDG?

- ◯ Yes
- ◯ No
- ◯ No, and to the best of my knowledge there are no NHS for CDG

* 20. What challenges did you find (please select ALL that apply)?

- [ ] Difficulty in patient recruitment
- [ ] Loss of patients to follow-up
- [ ] Elevated costs related to implementation and maintenance
- [ ] Other (please specify)

* 21. How important are patient registries and natural history studies for CDG therapy-driven research?

Please note that:
0 = I don’t think patient registries and natural history studies are important for CDG therapy-driven research.
5 = I think patient registries and natural history studies are essential for CDG therapy-driven research.

0	1	2	3	4	5
○	○	○	○	○	○

Workshop 3 – Emerging tools/methods to accelerate therapeutic discovery

* 22. Are you using or have you used Artificial Intelligence (AI) in your research?

- ○ Yes
- ○ No

* 23. Do you think Artificial Intelligence (AI) can help find new therapies for CDG?

- ○ Yes
- ○ No
- ○ I don't know

* 24. How can Artificial Intelligence (AI) help in drug discovery (please select ALL that apply)?

- [ ] It can combine data from different sources and reduce analysis costs and time
- [ ] It can be used to help physicians in diagnostics
- [ ] It can help search databases for new chemical compounds
- [ ] It can generate disease models to help drug development
- [ ] Other (please specify)
- [ ] It can predict drug side effects
- [ ] I don't think AI can help in drug discovery
- [ ] I don't know

* 25. How important are AI and drug repurposing for CDG therapy-driven research?

Please note that:
0 = I don’t think AI and drug repositioning are important for CDG therapy-driven research.
5 = I think patient AI and drug repositioning are essential for CDG therapy-driven research.

0	1	2	3	4	5
○	○	○	○	○	○

Workshop 4 – CDG models

* 26. Do you use CDG (*in vitro* and/or *in vivo*) disease models in your research?

- ○ Yes
- ○ No

* 27. Which models do you use (please select ALL that apply)?

- [ ] Commercial cell lines
- [ ] Immortalized patient-derived cell lines
- [ ] Patient-derived cell lines
- [ ] Embryonic stem cells
- [ ] iPSC cell line
- [ ] *S. cerevisae*
- [ ] *C. elegans*
- [ ] Mouse model
- [ ] Zebrafish model
- [ ] *D. melanogaster*
- [ ] Other (please specify)

* 28. Please select the major 3 obstacles for the development of disease models for CDG.

- [ ] Lack of interest from researchers
- [ ] Lack of interest from pharmaceutical industry
- [ ] High costs
- [ ] Lack of investment
- [ ] Lack of collaboration between researchers and pharmaceutical companies
- [ ] Lack of awareness about CDG
- [ ] Other (please specify)

* 29. Please select the 3 major fields of opportunities/action that can boost the development of disease models for CDG.

- [ ] Use of new genetic tools
- [ ] Cooperation between researchers
- [ ] Use of 3D disease models for research
- [ ] Guarantee the support of pharmaceutical industry
- [ ] Patients Association driven investment
- [ ] Other (please specify)

* 30. How important are disease models for CDG therapy-driven research?

Please note that:
0 = I don’t think disease models for CDG are important for CDG therapy-driven research.
5 = I think disease models for CDG are essential for CDG therapy-driven research.

0	1	2	3	4	5
○	○	○	○	○	○

Workshop 5 – Drug approval and access to patients

31. Are you aware of the following tools to speed drug approval?

	Yes	No	I don't know
PRIME (EMA)	◯	◯	◯
Conditional marketing authorization (EMA)	◯	◯	◯
Compassionate use (EMA)	◯	◯	◯
Fast Track (FDA)	◯	◯	◯
Accelerated Approval (FDA)	◯	◯	◯
Rare Pediatric Disease Priority Review Voucher Program (FDA)	◯	◯	◯
Orphan Drug Designation	◯	◯	◯

* 32. Do you think Patients have a role to play in the drug approval process?

◯ Yes

◯ No

◯ I don't know

U LISBOA | UNIVERSIDADE DE LISBOA

CDG & Allies – PPAIN
CDG & Allies - Professionals and Patient Associations International Network

4th World Conference CDG Questionnaire for Professionals – Assessing CDG needs and solutions for future therapies.

* 33. How can CDG patients participate in the drug approval process (please select ALL that apply)?

☐ Preparation of risk management plan summaries during the evaluation process

☐ Participation as members of EMA committees (e.g Scientific Advisory Group – SAG)

☐ Increasing disease awareness

☐ Lobbying for approval (e.g. eteplirsen for Duchenne muscular dystrophy)

☐ Other (please specify)

Workshop 6 – CDG diagnosis & clinical impact

* 34. On average and based on your experience, how long do you think a CDG diagnosis takes?

- ◯ 6 months or less
- ◯ 12 months or less
- ◯ Between 1 to 2 years
- ◯ Between 3 to 5 years
- ◯ Between 6 to 8 years
- ◯ 10 years or more
- ◯ Other (please specify)

* 35. Do you think CDG patients can play a role in improving disease diagnostics?

- ◯ Yes
- ◯ No
- ◯ I don't know

* 36. How can patients improve disease diagnostics (select ALL that apply)?

- ☐ Patients can report on symptoms not described in the literature
- ☐ Patients are experts in their own condition
- ☐ Patients and/or Patient Associations can contribute to educate medical professionals about their condition
- ☐ Other (please specify)

* 37. Please, from the following options, select the 3 major difficulties for CDG diagnosis.

- [ ] Lack of disease awareness
- [ ] Reduced number of patients
- [ ] Symptom variability
- [ ] Increase of the described CDG
- [ ] Reduced number of disease specialists
- [ ] Geographical dispersion of disease specialists
- [ ] Lack of disease management guidelines
- [ ] Lack of good disease biomarkers
- [ ] Lack of an education program in Rare Diseases for medical students and/or physicians
- [ ] Lack of patient participation and engagement in disease awareness
- [ ] Other (please specify)

* 38. Please, from the following options, select the 3 major improvements needed for CDG diagnosis.

- [ ] Better disease biomarkers
- [ ] Increase in disease awareness
- [ ] Better communication between patient and physicians
- [ ] Greater investment in research
- [ ] Increase in disease education for medical students and physicians
- [ ] Other (please specify)

* 39. Do you know what Patient Reported Outcome Measures (PROMs) are?

- ( ) Yes
- ( ) I have heard about PROMs but I don’t know what they are
- ( ) I have not heard about PROMs
- ( ) I don't know

* 40. Have you ever worked with PROMs?

- ( ) Yes
- ( ) No

* 41. How important are PROMs for CDG therapy-driven research?

Please note that:
0 = I don't think PROMs are important for CDG therapy-driven research.
5 = I think PROMs are essential for CDG therapy-driven research.

0	1	2	3	4	5
○	○	○	○	○	○

Workshop 7 – CDG therapies & clinical trials

* 42. Have you ever been involved (past and present situation) in the development of a clinical trial for CDG?

○ Yes

○ No

* 43. Have you involved Patients and/or Patient Representatives in the design of the clinical trial?

○ Yes

○ No

* 44. Have you informed the patients that participated in the clinical trial about the results?

○ Yes

○ No

* 45. Why not (please select ALL that apply)?

- [ ] The clinical trial is still ongoing
- [ ] I don’t think patients will understand the information
- [ ] The clinical trial was not successful and I’m afraid to disappoint the patients
- [ ] The clinical trial had some positive outcomes but it was not completely successful and I don’t want to give the patients false hopes
- [ ] I don't know
- [ ] Other (please specify)

* 46. How do you think CDG patients could participate in clinical trial development (please select ALL that apply)?

- [ ] Provide input on trial protocols and study design
- [ ] Help finalize eligibility criteria within the study protocol
- [ ] Provide qualitative feedback throughout trial participation that is fed back to the company/health care professionals/researcher
- [ ] Provide input for recruitment campaign
- [ ] Serve as peer advocate during the informed consent process
- [ ] I don't know
- [ ] Other (please specify)

* 47. Which are the main obstacles to therapy development for CDG (please, select ALL that applies)?

- [ ] Reduced number of patients
- [ ] High costs associated to drug development
- [ ] Lack of manpower
- [ ] The variability of symptoms and organs affected in CDG patients
- [ ] Lack of Natural History Studies to determine disease landscape
- [ ] I don't know
- [ ] Other (please specify)

* 48. Which are the main solutions to advance therapy development for CDG (please, select ALL that applies)?

- [ ] Cooperation between all stakeholders (researcher, medical professionals, patients)
- [ ] Increase disease awareness
- [ ] Development of better disease models
- [ ] The sharing of research results to avoid effort duplication
- [ ] Greater funding from industry
- [ ] I don't know
- [ ] Other (please specify)

* 49. How important are clinical trials for CDG therapy-driven research?

Please note that:
0 = I don’t think clinical trials are important for CDG therapy-driven research.
5 = I think clinical trials are essential for CDG therapy-driven research.

0	1	2	3	4	5
◯	◯	◯	◯	◯	◯

Workshop 8 – Tackling rare diseases challenges with international and interdisciplinary networks

* 50. Are you a member or have you ever participated in national/international interdisciplinary network(s)?

◯ Yes

◯ No

* 51. Are you aware of the following networks for Rare Diseases?

	Yes and I find it useful	Yes but I don't find it useful	No	I don't know
European Reference Network for Hereditary Metabolic Disorders (MetabERN)	◯	◯	◯	◯
European Organization for Rare Diseases (EURORDIS)	◯	◯	◯	◯
International Rare Disease Research Consortium (IRDiRC)	◯	◯	◯	◯
National Organization for Rare Disorders (NORD)	◯	◯	◯	◯
Rare Diseases Clinical Research Network (RDCRN)	◯	◯	◯	◯
Share4Rare	◯	◯	◯	◯

* 52. Do you think social media could help researchers and medical professionals disseminate knowledge about CDG?

- ◯ Yes
- ◯ No
- ◯ I don't know

* 53. Please rate the following sentences regarding social media and rare diseases/CDG.

	Completely agree	Partially agree	Partially disagree	Completely disagree	I don't know
Social media increases disease awareness	○	○	○	○	○
Social media helps in patient recruitment, retention and monitoring in clinical trials	○	○	○	○	○
Social media assists researchers to better understand patients and their condition	○	○	○	○	○
Social media facilitates information sharing	○	○	○	○	○
Social media could assist in clinical trials design	○	○	○	○	○
In order to use social media in clinical trials companies must assure patient privacy and data security	○	○	○	○	○
Social media may contain inaccurate information that will mislead patients	○	○	○	○	○
Social media can exclude patients who don’t have access to such technology	○	○	○	○	○
Researcher/medical professionals should work with Patient Associations to provide reliable information to be shared on social media	○	○	○	○	○

Thank you for completing your questionnaire!

Thank you for taking the time to participate in our questionnaire. We truly value the information you have provided. Your participation is vital in helping CDG community to raise CDG awareness.

CDG

Awareness
