# Supplementary material for: Stakeholders’ views on drug development: the congenital disorders of glycosylation community perspective
Source: Orphanet J Rare Dis. 2022 Jul 30;17:303. doi: 10.1186/s13023-022-02460-0 (PMC9338569; doi:10.1186/s13023-022-02460-0)
Supplement: Supplementary file 13 — Additional file 13. E-survey - version adapted to families. [file 13023_2022_2460_MOESM13_ESM.pdf]

4th World Conference CDG Questionnaire for Patients & Caregivers – Assessing CDG needs and solutions for future therapies

4th World Conference CDG Questionnaire - Instructions and informed consent for participants

4th World Conference CDG Questionnaire – Assessing CDG needs and solutions for future therapies

Before deciding or not to participate, it's important that you understand the purpose of this study.
By agreeing to participate, you are agreeing with the terms and conditions explained in this document.

About the questionnaire
The full comprehension of the therapy landscape for CDG requires the consultation and involvement of all stakeholders. Also, it is imperative to educate and empower the CDG Patient Community, besides promoting interaction and communication among all members of the community. This questionnaire intends to assess the level of knowledge and awareness about various aspects related to the drug development process among the Congenital Disorders of Glycosylation (CDG) community (patients, family members, caregivers, etc.).

The main goals of this questionnaire are to:

- Pool knowledge on CDG therapy research and drug development process from all the CDG community stakeholders;
- Identify the knowledge gaps and needs among the CDG Community;
- Evaluate and compare the perspectives of CDG Patients/Caregivers vs Professionals;
- Identify the possible common solutions to increase knowledge regarding drug development process.

The Team
This project has been developed by:

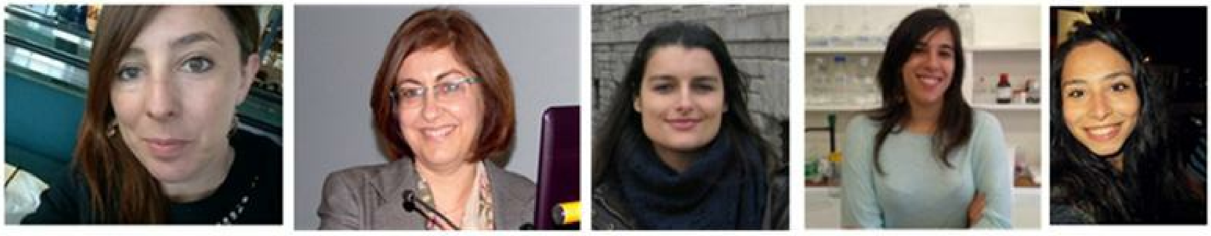

Dr. Vanessa Ferreira | Dr. Luísa Barros | Dr. Sandra Brasil | Rita Francisco (PhD student) | Carlota Pascoal (PhD student)

This **questionnaire** will be available **only in English** and should **take about 15 – 20 minutes to complete**.

NOTE THAT you don't have to fill in the entire questionnaire all at once. You can stop at any point, how many times you need and finish it when it's convenient to you as long as it is done within the deadline of the project. To ensure that you can return to the question you were at when you left the survey, MAKE SURE you use the SAME DEVICE (e.g. computer or phone) and INTERNET BROWSER (e.g. Google Chrome).

Who can participate?
You can participate if you are a family member/caregiver of a CDG patient(s) or if you are a CDG patient yourself (in both cases you need to be 18 years old or older).
Participation is voluntary.
At any stage of the questionnaire you can stop filling it out. You don't need to justify your decision and that won't affect you in anyway.

Will my information be published?
This survey is anonymous. We only ask for the necessary information to analyze the results. No personal information will be published. Data will be analysed and the results of this survey will be presented at the **4th World Conference for CDG** and used for further scientific publications that will be made available on APCDG (www.apcdg.com) and CDG & Allies – PPAIN (www.researchcdg.com) websites.

Personal data collection abides the Directive (EU) 2016/679 from 27th April 2016.

Who can I contact if I have doubts?
If you need additional information about the questionnaire, language or clarifications on the content, please do not hesitate to write to the following address: sd.brasil@fct.unl.pt

* 1. **ELECTRONIC CONSENT:** Please select your choice below.

Clicking on the "agree" button below indicates that:

· **You have read and understood the previous information,**

· **You voluntarily agree to participate,**

· **You are a patient, patient family member and/or caregiver.**

If you wish to participate in the research study, please accept participation by clicking on the "agree" button.

◯ Agree

◯ Disagree

* 2. Are you a...

- ◯ CDG Patient
- ◯ CDG Sibling
- ◯ CDG Parent
- ◯ CDG Grandparent
- ◯ Other (please specify)

* 3. What is your education level?

- ◯ No formal education
- ◯ High school diploma
- ◯ College degree
- ◯ Bachelor’s degree
- ◯ Master’s degree
- ◯ Professional degree
- ◯ Doctorate degree
- ◯ Other (please specify)

* 4. In which country do you live in?

* 5. Do you collaborate or are involved with a Patient Association (e.g. board member, Patient Advocate, volunteer)?

- ◯ Yes
- ◯ No

Workshop 1 – Research and development

* 6. On average, how long do you think it takes to develop a therapy and for patients to have access to it?

- ◯ 1 year
- ◯ 5 year
- ◯ 7 year
- ◯ 10 year
- ◯ 12 year
- ◯ 15 year
- ◯ 20 year
- ◯ Other (please specify)

* 7. Have you as a Patient/Family member ever been a research participant?

- ◯ Yes
- ◯ No
- ◯ I don't know

4th World Conference CDG Questionnaire for Patients & Caregivers – Assessing CDG needs and solutions for future therapies

* 8. What has worked well (please select ALL that apply)?

- ☐ I was able to inform researchers about my experience as a patient
- ☐ I felt useful contributing to the advance of research in CDG
- ☐ I think I was able to improve the research project with my views and experience
- ☐ I was able to set up research projects
- ☐ Other (please specify)

* 9. What has not worked well (please select ALL that apply)?

- ☐ Communication was hard sometimes due to the scientific language
- ☐ I don’t think researchers were truly interested in my point of view
- ☐ I was not able to explain my point of view
- ☐ My input was not taken into account
- ☐ Other (please specify)

* 10. Given the opportunity, would you be willing to participate in research as a Patient/Family member?

- ◯ Yes
- ◯ No
- ◯ I don't know

* 11. Do you know what a biomarker is?

- ◯ Yes
- ◯ I have heard about biomarkers but I don’t know what they are
- ◯ I have not heard about biomarkers
- ◯ I don't know

4th World Conference CDG Questionnaire for Patients & Caregivers – Assessing CDG needs and solutions for future therapies

* 12. Do you know if there are specific biomarkers for CDG?

- ◯ Yes
- ◯ No
- ◯ I don't know

4th World Conference CDG Questionnaire for Patients & Caregivers – Assessing CDG needs and solutions for future therapies

* 13. Where have you learned about biomarkers for CDG?

- ◯ Internet (Google search)
- ◯ From a Clinician/ Researcher
- ◯ Patient Associations
- ◯ Other (please specify)
- ◯ Social media
- ◯ Scientific Conference

[ ]

* 14. How important are biomarkers for CDG therapy-driven research?

Please note that:

0 = I don’t think biomarkers are important for CDG therapy-driven research.

5 = I think biomarkers are essential for CDG therapy-driven research.

0	1	2	3	4	5
◯	◯	◯	◯	◯	◯

* 15. Do you know what biobanks are?

- ◯ Yes
- ◯ I have heard about biobanks but I don’t know what they are
- ◯ I have not heard about biobanks
- ◯ I don't know

* 16. How important are biobanks for CDG therapy-driven research?

Please note that:

0 = I don’t think biobanks are important for CDG therapy-driven research.

5 = I think biobanks are essential for CDG therapy-driven research.

0	1	2	3	4	5
◯	◯	◯	◯	◯	◯

Workshop 2 – Tools for preclinical and clinical drug development and approval

* 17. Do you think Patients have an active role (e.g. as part of EMA and/or FDA committees) to play in the drug development process?

- ◯ Yes
- ◯ No
- ◯ I don't know

* 18. How familiar are you with the drug development process?

- ◯ Very familiar
- ◯ Familiar
- ◯ Slightly familiar
- ◯ Not familiar
- ◯ I have never heard about “drug development process"
- ◯ I don't know

* 19. Are you aware of the following initiatives to ensure Patient participation in drug development?

	I'm aware and have participated	I'm aware but have never participated	I'm not aware	I don't know
Patient-focused drug development (PFDD) from FDA	◯	◯	◯	◯
Patient Engagement Collaborative Establishment from FDA	◯	◯	◯	◯
Patients' and Consumers' Working Party (PCWP) from EMA	◯	◯	◯	◯
Committee for Orphan Medicinal Products (COMP) from EMA	◯	◯	◯	◯
European Public Assessment Reports (EPAR) from EMA	◯	◯	◯	◯

* 20. Do you know what a Patient Registry is?

◯ Yes

◯ I have heard about Patient Registries but I don't know what they are

◯ I have not heard about Patient Registries

◯ I don't know

* 21. Have your clinical data ever been collected for a Patient Registry?

◯ Yes

◯ No

◯ I don't know

* 22. Patient Registries can be funded and/or managed by (please select ALL that apply):

☐ Government agencies

☐ Non-profit organizations

☐ Clinics

☐ Industry (e.g. pharma companies)

☐ I don't know

* 23. Do you know what a Natural History Study is?

- ◯ Yes
- ◯ I have heard about Natural History Studies but I don’t know what they are
- ◯ I have not heard about Natural History Studies
- ◯ I don't know

4th World Conference CDG Questionnaire for Patients & Caregivers – Assessing CDG needs and solutions for future therapies

* 24. Have you ever participated in a Natural History Study?

- ◯ Yes
- ◯ No
- ◯ I don't know

* 25. Are Natural History Studies the same as or equivalent to Patient Registries?

- ◯ Yes
- ◯ No
- ◯ I don't know

* 26. Natural History Studies allow to (please select ALL that apply):

- ☐ Track the course of disease over time
- ☐ Identify demographic, genetic, environmental and other variables that correlate with disease outcomes in the absence of treatment
- ☐ Create better care and practices
- ☐ Define research priorities
- ☐ Develop of clinical trials
- ☐ I don't know

* 27. Which do you think are the major difficulties for Patient Registries and Natural History Studies in CDG? Please, select all the options that apply for patient registries and natural history studies.

	Patient registries	Natural History Studies
Reduced number of patients	☐	☐
Geographical dispersion of patients and/or medical professionals	☐	☐
Costs associated with setting-up and maintenance	☐	☐
Lack of information sharing among researchers and/or medical professionals	☐	☐
Lack of interest of pharmaceutical companies	☐	☐
Lack of information regarding the benefits of participation	☐	☐
Lack of patient engagement and participation	☐	☐
The burden related to giving medical information and keeping that information updated over time	☐	☐
I don't know	☐	☐

Other (please specify)

* 28. How important are patient registries and natural history studies for CDG therapy-driven research?

Please note that:
0 = I don’t think patient registries and natural history studies are important for CDG therapy-driven research.
5 = I think patient registries and natural history studies are essential for CDG therapy-driven research.

0	1	2	3	4	5
◯	◯	◯	◯	◯	◯

Workshop 3 – Emerging tools/methods to accelerate therapeutic discovery

* 29. Have you ever heard about drug repositioning/repurposing?

◯ Yes

◯ I have heard about drug repositioning/repurposing but I don’t know what it is

◯ I have not heard about drug repositioning/repurposing

◯ I don't know

* 30. Do you think drug repositioning is important for the discovery of new therapeutic approaches for CDG?

◯ Yes

◯ No

◯ I don't know

* 31. Do you know what Artificial Intelligence (AI) is?

◯ Yes

◯ I have heard about AI but I don’t know what it is

◯ I have not heard about AI

◯ I don't know

* 32. Do you think Artificial Intelligence (AI) can help find new therapies for CDG?

- ◯ Yes
- ◯ No
- ◯ I don't know

* 33. How can Artificial Intelligence (AI) help in drug discovery (please select ALL that apply)?

- ☐ It can combine data from different sources and reduce analysis costs and time
- ☐ It can generate disease models to help drug development
- ☐ It can be used to help physicians in diagnostics
- ☐ It can predict drug side effects
- ☐ It can help search databases for new chemical compounds
- ☐ I don't know
- ☐ Other (please specify)

* 34. How important are AI and drug repurposing for CDG therapy-driven research?

Please note that:
0 = I don't think AI and drug repositioning are important for CDG therapy-driven research.
5 = I think patient AI and drug repositioning are essential for CDG therapy-driven research.

0	1	2	3	4	5
◯	◯	◯	◯	◯	◯

Workshop 4 – CDG models

* 35. Do you think disease models (e.g. animal models) for CDG research are essential for the development of a therapy?

- ◯ Yes
- ◯ No
- ◯ I don't know

* 36. Are you familiar with the disease models available for research in CDG?

- ◯ Yes
- ◯ No
- ◯ I don't know

* 37. Do you know the difference between *in vitro* and *in vivo* disease models?

- ◯ Yes
- ◯ No
- ◯ I don't know

* 38. Please select the major 3 obstacles for the development of disease models for CDG

- ☐ Lack of interest from researchers
- ☐ Lack of interest from pharmaceutical industry
- ☐ High costs
- ☐ Lack of investment
- ☐ Lack of collaboration between researchers and pharmaceutical companies
- ☐ Lack of awareness about CDG
- ☐ Other (please specify)

* 39. How important are disease models for CDG therapy-driven research?

Please note that:
0 = I don't think disease models for CDG are important for CDG therapy-driven research.
5 = I think disease models for CDG are essential for CDG therapy-driven research.

0	1	2	3	4	5
◯	◯	◯	◯	◯	◯

Workshop 5 – Drug approval and access to patients

* 40. To the best of your knowledge, is there any kind of therapy/treatment for your (the patient) CDG type?

- ◯ Yes
- ◯ No
- ◯ I don't know

* 41. Do you have access to it (i.e. is the patient taking it)?

- ◯ Yes
- ◯ No
- ◯ I don't know

* 42. Please name the therapy/treatment.

[ ]

* 43. Can you specify the reason?

- ◯ Too expensive
- ◯ Not available in my country
- ◯ It has not been developed yet
- ◯ I don't know
- ◯ Other (please specify)

[ ]

* 44. How familiar do you think you are with the drug approval process?

- ◯ Very familiar
- ◯ Familiar
- ◯ Slightly familiar
- ◯ Not familiar
- ◯ I have never heard about “drug development process"
- ◯ I don't know

* 45. Do you think Patients have a role to play in the drug approval process?

- ◯ Yes
- ◯ No
- ◯ I don't know

* 46. Do you know what the Orphan Drug designation is?

- Yes
- I have heard about Orphan drug designation but I don’t know what it is
- I have not heard about Orphan drug designation
- I don't know

Workshop 6 – CDG diagnosis & clinical impact

* 47. How long did it take for you (the patient) to get the CDG diagnosis?

- 6 months or less
- 12 months or less
- Between 1 to 2 years
- Between 3 to 5 years
- Between 6 to 8 years
- 10 years or more
- Other (please specify)

* 48. How important was it for you to get a diagnosis?

- Very important
- Important
- More or less important
- Not important
- I don't know

* 49. Before getting the CDG diagnosis, did you (the patient) experienced a misdiagnosis?

- Yes
- No
- I don't know

* 50. Which diagnosis was made? Please specify.

* 51. Please, from the following options, select the 3 major difficulties for CDG diagnosis.

- [ ] Lack of disease awareness
- [ ] Reduced number of patients
- [ ] Geographical dispersion of patients
- [ ] Reduced number of disease specialists
- [ ] Geographical dispersion of disease specialists
- [ ] Lack of disease management guidelines
- [ ] Lack of good disease biomarkers
- [ ] Lack of an education program in Rare Diseases for medical students and/or physicians
- [ ] Lack of patient participation and engagement in disease awareness
- [ ] Other (please specify)

* 52. Please, from the following options, select the 3 major improvements for CDG diagnosis.

- [ ] Better disease biomarkers
- [ ] Increase in disease awareness
- [ ] Better communication between patient and physicians
- [ ] Greater investment in research
- [ ] Increase in disease education for medical students and physicians
- [ ] Other (please specify)

* 53. Do you know what Patient Reported Outcome Measures (PROMs) are?

- ( ) Yes
- ( ) I have heard about PROMs but I don’t know what they are
- ( ) I have not heard about PROMs
- ( ) I don't know

* 54. How important are PROMs for CDG therapy-driven research?

Please note that:
0 = I don’t think PROMs are important for CDG therapy-driven research.
5 = I think PROMs are essential for CDG therapy-driven research.

0	1	2	3	4	5
○	○	○	○	○	○

Other (please specify)

Workshop 7 – CDG therapies & clinical trials

* 55. Do you know what a clinical trial is?

- ○ Yes
- ○ I have heard about clinical trials but I don’t know what they are
- ○ I have not heard about clinical trials
- ○ I don't know

* 56. Have you (the CDG patient) ever participated in a clinical trial for CDG?

- ○ Yes
- ○ Yes, and I (the patient) am (is) currently taking part in a clinical trial
- ○ No
- ○ I don't know

* 57. Why haven’t you (the patient) participated (please select ALL that apply)?

- [ ] I was not aware of any clinical trial for my (the patient) CDG type
- [ ] The clinical trial site is too far from my home and I don’t have financial possibilities to go there
- [ ] I (the patient) didn’t fit the inclusion criteria of the study
- [ ] The medical insurance didn’t cover the expenses
- [ ] The clinical trial site is too far from my home town
- [ ] The disease has stabilized and I don’t think a clinical trial would be useful
- [ ] Other (please specify)

* 58. How were you (the patient) recruited to participate in the clinical trial?

- ( ) Through a physician
- ( ) Through social media
- ( ) Through a Patient Organization
- ( ) Through a researcher
- ( ) Other (please specify)

* 59. Were you involved in the design of the clinical trial (ex. elaboration and/or revision of the outcome measures, the informed consent documents, the dissemination and recruitment strategy)?

- ( ) Yes
- ( ) No
- ( ) No, and I didn’t know patients/participants/families could be involved in the design of a clinical trial

* 60. Were you informed of the results of the clinical trial in which you (the patient) have participated?

- ( ) Yes
- ( ) No, and the trial has already finished
- ( ) No, but the trial is still ongoing
- ( ) I don't know

* 61. How do you think CDG patients could participate in clinical trial development (please select ALL that apply)?

- [ ] Provide input on trial protocols and study design
- [ ] Help finalize eligibility criteria within the study protocol
- [ ] Provide qualitative feedback throughout trial participation that is fed back to the company/health care professionals/researcher
- [ ] Provide input for recruitment campaign
- [ ] Serve as peer advocate during the informed consent process
- [ ] I don't know
- [ ] Other (please specify)

* 62. How important are clinical trials for CDG therapy-driven research?

Please note that:
0 = I don’t think clinical trials are important for CDG therapy-driven research.
5 = I think clinical trials are essential for CDG therapy-driven research.

0	1	2	3	4	5
○	○	○	○	○	○

Workshop 8 – Tackling rare diseases challenges with international and interdisciplinary networks

* 63. Are you a member or have you ever participated as a Patient Representative in national/international interdisciplinary networks

○ Yes

○ No

* 64. Are you aware of the following networks for Rare Diseases?

	Yes and I find it useful	Yes but I don´t find it useful	No	I don't know
European Reference Network for Hereditary Metabolic Disorders (MetabERN)	◯	◯	◯	◯
European Organization for Rare Diseases (EURORDIS)	◯	◯	◯	◯
International Rare Disease Research Consortium (IRDiRC)	◯	◯	◯	◯
National Organization for Rare Disorders (NORD)	◯	◯	◯	◯
Rare Diseases Clinical Research Network (RDCRN)	◯	◯	◯	◯
Share4Rare	◯	◯	◯	◯

Other (please specify)

[ ]

* 65. Have you ever used social media (ex. Facebook, Twitter, LinkedIN) to solve questions about CDG?

◯ Yes

◯ No

◯ I don't know

* 66. Do you think social media could help researchers and medical professionals disseminate knowledge about CDG?

- Yes
- No
- I don't know

* 67. Do you think researchers and physicians should use social media to share results and disseminate knowledge about CDG?

- Yes
- No
- I don't know

* 68. Please rate the following sentences regarding social media and rare diseases/CDG.

	Completely agree	Partially agree	Partially disagree	Completely disagree	I don’t know
Social media increases disease awareness	○	○	○	○	○
Social media helps in patient recruitment, retention and monitoring in clinical trials	○	○	○	○	○
Social media assists researchers to better understand patients and their condition	○	○	○	○	○
Social media facilitates information sharing	○	○	○	○	○
Social media could assist in clinical trials design	○	○	○	○	○
In order to use social media in clinical trials companies must assure patient privacy and data security	○	○	○	○	○
Social media may contain inaccurate information that will mislead patients	○	○	○	○	○
Social media can exclude patients who don’t have access to such technology	○	○	○	○	○

Thank you for completing your questionnaire!

Thank you for taking the time to participate in our questionnaire. We truly value the information you have provided. Your participation is vital in helping CDG community to raise CDG awareness.

CDG

Awareness
